# Asymmetric Michael Addition Organocatalyzed by α,β-Dipeptides under Solvent-Free Reaction Conditions

**DOI:** 10.3390/molecules22081328

**Published:** 2017-08-10

**Authors:** C. Gabriela Avila-Ortiz, Lenin Díaz-Corona, Erika Jiménez-González, Eusebio Juaristi

**Affiliations:** 1 Departamento de Química, Centro de Investigación y de Estudios Avanzados, Avenida IPN 2508, Ciudad de México 07360, Mexico; gabriela@relaq.mx (C.G.A.-O.); lenindc8@relaq.mx (L.D.-C.); erika@relaq.mx (E.J.-C.); 2 El Colegio Nacional, Luis González Obregón 23, Centro Histórico, Ciudad de México 06020, Mexico

**Keywords:** peptides, Michael addition, asymmetric organocatalysis, solvent-free reactions

## Abstract

The application of six novel α,β-dipeptides as chiral organocatalysts in the asymmetric Michael addition reaction between enolizable aldehydes and *N*-arylmaleimides or nitroolefins is described. With *N*-arylmaleimides as substrates, the best results were achieved with dipeptide **2** as a catalyst in the presence of aq. NaOH. Whereas dipeptides **4** and **6** in conjunction with 4-dimethylaminopyridine (DMAP) and thiourea as a hydrogen bond donor proved to be highly efficient organocatalytic systems in the enantioselective reaction between isobutyraldehyde and various nitroolefins.

## 1. Introduction

Organocatalysis has become a powerful method in organic synthesis as can be appreciated by the rapidly increasing number of publications on this topic [1,2,3,4,5,6,7,8,9,10,11,12,13,14]. Of special interest is the implementation of organocatalytic processes that are environmentally friendly [15,16,17]. One way to achieve this goal is by employing alternative activation techniques such as microwaves, ultrasound irradiation and mechanochemistry [18,19,20]. Furthermore, removal of the solvent in the reaction leads to a reduction of the generated waste [21,22,23,24,25]. There are several examples in literature of organocatalytic reactions in the absence of solvent [26,27,28,29,30,31,32,33].

**Scheme 1:**
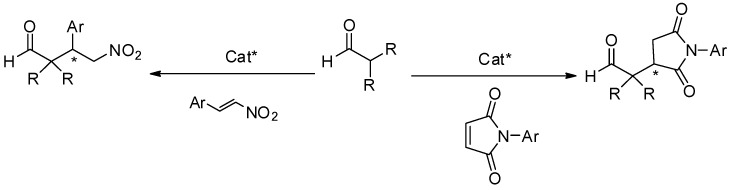
Asymmetric Michael addition reaction of aldehydes to nitroolefins and *N*-arylmaleimides as acceptor substrates. Cat*, chiral catalyst; *, chiral carbon; Ar, aryl.

In this context, the Michael addition reaction is a particularly powerful method for C–C bond formation. Nevertheless, the enantioselective Michael addition reaction between enolizable aldehydes and maleimides [34,35,36,37,38,39,40,41,42,43,44,45,46,47] or nitroolefins [28,29,30,48,49,50,51,52,53] has rarely been studied in relation to the reaction with cyclohexanone (Scheme 1).

**Figure 1:**
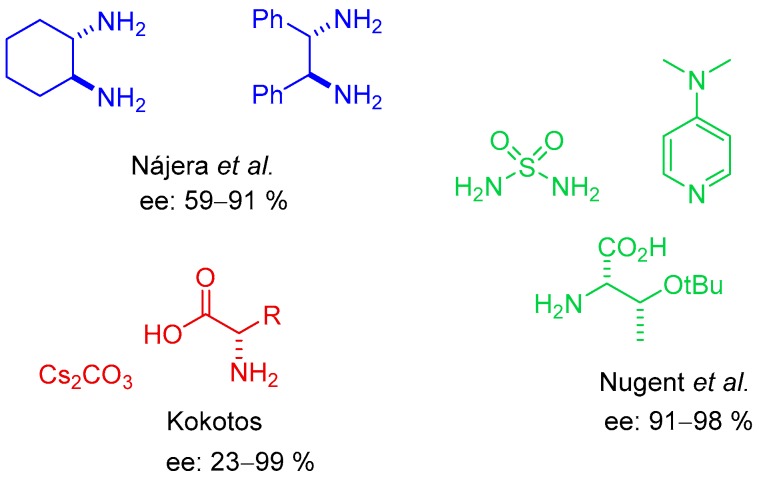
Examples of organocatalysts and additives employed in the Michael addition reaction of aldehydes to maleimides and/or nitroolefins, reported by Nájera et al. [55,56], Kokotos [57], and Nugent et al. [58,59]. ee, enantiomeric excess.

In 2007, Cordova’s research group [54] was the first to study the addition reaction of isobutyraldehyde to maleimides. More recently, Nájera’s group [55,56] studied the reaction employing several chiral 1,2-diamines as catalysts, while Kokotos used different α- or β-amino acids as organocatalysts in the presence of CsCO_3_ [57]. Finally, Nugent [58,59] examined the use of isoleucine or threonine as organocatalyst in the presence of a hydrogen bond donor (sulfamide) and a base (4-dimethylaminopyridine, DMAP) (Figure 1).

**Scheme 2:**
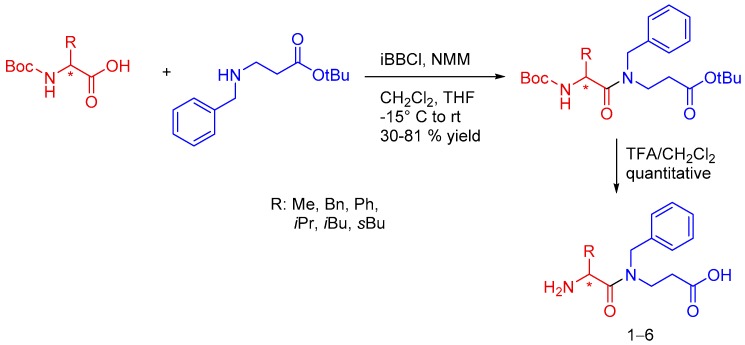
Synthetic route to the α,β-peptides of interest in the present work. Me, methyl; Bn, benzyl; Ph, phenyl; *i*Pr, isopropyl; *i*Bu, isobutyl; *s*Bu, *sec*-butyl; iBBCl, isobutyl chloroformate; THF, tetrahydrofuran; rt, room temperature; TFA, trifluoroacetic acid.

A few years ago, our research group reported the synthesis of a family of α,β-dipeptides **1**–**6**, which were used as precursors in the synthesis of 7-membered heterocyclic type ([1,4]-diazepin-2,5-diones, that is homodiketopiperazines) derivatives (Scheme 2) [60]. The desired α,β-dipeptides were readily obtained by the coupling of protected β-alanine (β-Ala) and the *N*-*tert*-Butoxycarbonyl (*N*-Boc) protected amino acid, followed by the removal of the protecting groups. Yields for the coupling step went from moderate to good, depending on the side chain present in the α-amino acid. Deprotection of these peptides with trifluoroacetic acid and subsequent isolation using an ion exchange column with Dowex resin afforded the desired α,β-dipeptides **1**–**6**.

Given the proven efficiency of small peptides in asymmetric organocatalysis [61,62,63], and having access to the α,β-peptides depicted in Scheme 2, we deemed it of interest to test their potential as catalysts in the asymmetric Michael addition reaction. In this regard, β-amino acids have been used successfully as organocatalysts in asymmetric Michael addition reactions [57,64], thus their incorporation in dipeptidic organocatalysts was anticipated to result in more efficient activation modes. Furthermore, although β-amino acids are not found as frequently in nature as their α-analogs, they represent a very important research area in organic synthesis since the 1990s [65,66]. Of great relevance to the present work, it has been discovered that the incorporation of β-amino acids in peptides induces significant conformational changes in the resulting foldamers [67,68,69], which may prove beneficial in boosting the enantioinduction in the asymmetric Michael additions of interest here.

## 2. Results

**Figure 2:**
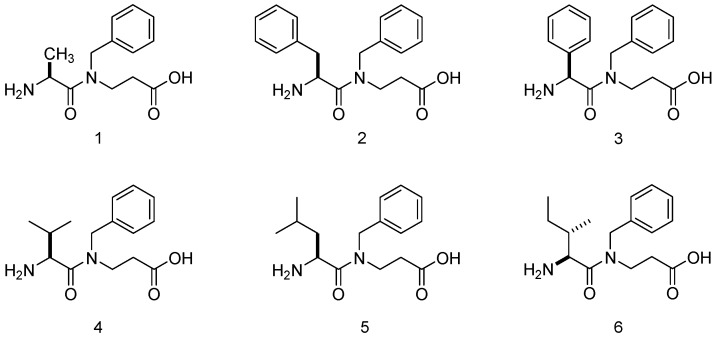
Dipeptides **1**–**6** examined as potential chiral organocatalysts.

α,β-Dipeptides **1**–**6** were examined as potential catalysts in the present work (Figure 2). It is important to mention that the β-Ala residue gives certain advantages to these molecules in comparison to their α,α-analogs. In particular, the phenylalanine-glycine (Phe-Gly) dipeptide was synthetized in the present work in order to compare its catalytic activity. In the event, the synthetic route resulted in poor yields (see Supplementary Materials) owing to glycine’s low solubility [70].

Initially, we conducted Michael additions of isobutyraldehyde to *N*-phenylmaleimide in the absence of solvent (neat), as shown in Table 1. It is important to mention that it became evident that a base is required for the reaction to take place. Based on Kokotos’s observations [57], KOH was chosen as the base additive in this system. It can be observed that the peptides which afforded better results in terms of yield and selectivity were phenylalanine-β-alanine (Phe-β-Ala, **2**), leucine-β-alanine (Leu-β-Ala, **5**) and isoleucine-β-alanine (Ileu-β-Ala, **6**) (Table 1). As peptide **2** can be synthesized in a higher yield [60], this dipeptide was selected for further optimization experiments. α,α-Dipeptide (Phe-Gly) gave a poor yield of Michael adduct with a lack of enantioselectivity (59% yield, 52:48 enantiomeric ratio (er). See Supplementary Materials).

With dipeptide **2** as the representative chiral catalyst, we proceeded to optimize the reaction in terms of several key parameters. The first parameter that was evaluated was catalyst loading. A screening was performed varying the concentration of catalyst from 1 to 25 mol %, finding that the optimum amount of dipeptide **2** corresponds to 10 mol % (Table 2). Similar observations have been made by Berkessel, Gröger and co-workers in related systems [70].

Next, various hydroxides, carbonates as well as several amines were tested as base additives. These bases were used in equimolar amounts relative to catalyst **2** (10 mol %). In Table 3, it can be appreciated that hydroxides are the most efficient base additives, leading to better yields and stereoselectivities. Among them, sodium hydroxide afforded higher enantioselectivity (Table 3, entry 3).

Furthermore, the reaction was examined in solution, exploring different solvents as reaction mediums. It transpired that only dichloromethane afforded results comparable in yield and selectivity (77% yield, 88:12 enantiomeric ratio. Table S1 of Supporting Information) relative to the reaction carried out under neat conditions. No reaction took place with the rest of the solvents that were examined (see Supplementary Materials). Taking into account the above observations, it was decided to continue the work in the absence of solvent to further promote processes which are friendlier to the environment [15,16,17,18,19,20,21,22,23,24,25]. In this regard, the amount of aldehyde substrate was optimized at this point. To our benefit, the reaction may proceed well with only 5.5 equivalents of isobutyraldehyde, which is the minimum quantity required to have a homogeneous reaction mixture—with less equivalents, the reaction becomes too slow.

It may be argued that isobutyraldehyde being used in excess in the reaction (5.5 equivalents relative to the *N*-phenyl maleimide substrate) actually acts as a as solvent and reagent. Nevertheless, this is one aspect of the area of green chemistry where there is clearly no consensus. In particular, according to the philosophy of Sheldon [21], who states “the best solvent is no solvent”, one strategy for the development of more environmentally friendly protocols involves solvent-free reactions. In some cases, this has been achieved using an excess (up to 20 equivalents) of a liquid reagent [31,71,72].

The potential influence of other additives, in particular hydrogen bond donors such as urea, thiourea and sulfamide, was also examined; however, these additive acids did not lead to any significant improvement of the stereoselectivity of the reaction. A similarly disappointing observation was made when the reaction was performed at a low temperature: the reaction became too slow, and the enantioselectivity did not actually improve (Table S2 of Supplementary Materials).

Once the reaction conditions had been optimized, the scope of the reaction was evaluated with different maleimides as electrophilic substrates. Table 4 summarizes the results. It can be observed that *N*-donor substituents in the aromatic group on the *N*-substituted maleimide cause a decrease in the reactivity, which results in poor reaction yields.

The potential of dipeptides **1**–**6** in the Michael addition to nitroolefins was also studied. Initially, the optimized conditions of the reaction with *N*-substituted maleimides were employed, but surprisingly the reaction did not proceed. Therefore, dipeptides **1**–**6** were tested under the conditions reported by Nugent et al. [58,59] who employ DMAP and a hydrogen bond donor (to ensure the proximity of both substrates) as additives (Table 5). Catalysts **4** and **6** provided the best results and were used in subsequent studies.

With dipeptide **6** as the catalyst, the effect of catalyst loading was evaluated. Examination of Table 6 shows that the most suitable catalyst load is 10 mol % (entry 3). It is important to note that as in the case of the reaction with maleimides, the use of solvent afforded the desired products in lower yield and decreased stereoselectivity. It was observed that both dichloromethane (DCM) and water solvent gave the desired product with high selectivity (93:7 and 94:6 er, respectively, Table S3 of Supplementary Materials). Finally, yields went from moderate to good (64% and 81%, respectively, Table S3 of Supplementary Materials). Again, the amount of aldehyde was optimized to 5.5 equivalents in order to use the minimum quantity to perform the reactions maintaining the same results in yield and selectivity.

Following the methodology reported by Nugent and co-workers [58,59], an analysis of the reaction was undertaken in the presence of different additives. Specifically, three different hydrogen bond donors were tested: urea, thiourea and sulfamide. The results turned out to be slightly better with thiourea, which is also readily accessible. Similar observations were made with dipeptides **4** and **6** as catalysts. The potential effect of temperature was also studied. Nevertheless, at low temperatures (−15 °C and +2 °C) the required reaction times turned out to be too long. Thus, it was concluded that the best reaction conditions correspond to the employment of 10 mol % of catalyst in the presence of equimolar amounts of urea or thiourea and DMAP as additives, at ambient temperature, and in the absence of solvent. Table 7 summarizes the observations derived from this evaluation.

Once the reaction conditions had been optimized, catalysts **4** and **6** were used to carry out Michael addition reactions with different substrates, in order to establish the scope of the reaction. Table 8 summarizes the results. Generally, both **4** and **6** catalysts provided the desired products with similarly high enantiomeric purity. It is important to note that electron withdrawing groups (EWG) conducts to lower yields in comparison to electron donating groups (EDG). The most dramatic effect was produced with 2-Br substituted nitroolefin, which gave the lowest yield but despite that, the product’s stereoselectivity was good (Table 8, essay 2).

**Scheme 3:**
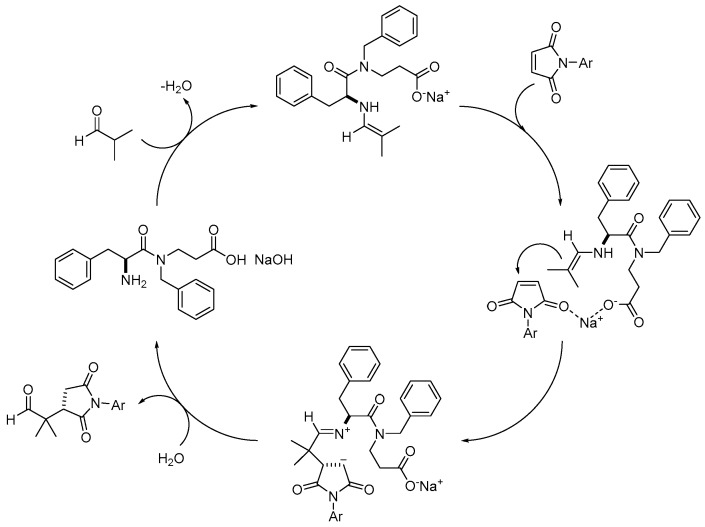
Proposed mechanism for the Michael addition reaction of isobutyraldehyde to *N*-arylmaleimides catalysed by peptide **2** in the presence of sodium hydroxide as a base.

Based on the mechanistic observations reported by Nugent [58,59], we propose a plausible mechanism for the reaction of isobutyraldehyde with both maleimides and *trans*-β-nitrostyrenes (Scheme 3).

## 3. Discussion

For the Michael addition reaction of aldehydes to *N*-arylmaleimides, the first step should correspond to enamine formation. The sodium carboxylate that is generated by the hydroxide base orients the approach of the electrophile, the Na^+^ cation acting as a bridging Lewis acid. Formation of the new C–C bond takes place with the concomitant creation of the chiral center. Finally, hydrolysis of the iminium ion intermediate gives the desired product (Scheme 3).

**Scheme 4:**
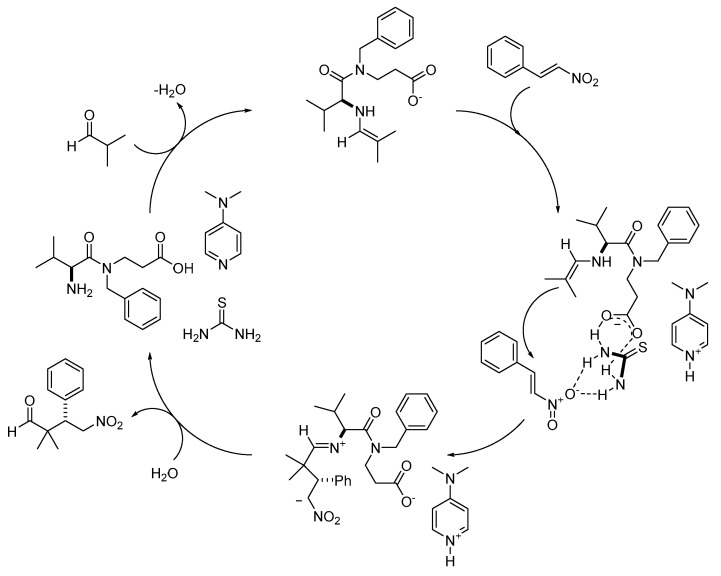
Proposed mechanism for the Michael addition reaction of isobutyraldehyde to *trans*-β-nitrostyrene catalysed by peptide **4** in the presence of DMAP and thiourea as the hydrogen bond donor.

In the case of enolate addition to nitroolefins, the first step should be an acid-base reaction between the dipeptide and DMAP, followed by enamine formation. In this case, the resulting intermediate step is activated by the thiourea molecule, which helps orient the approach of the nitroolefin to the enamine. Final hydrolysis gives the Michael adduct (Scheme 4).

## 4. Materials and Methods

*Methyl ((benzyloxy)carbonyl)-L-phenylalanylglycinate* (**21**)**.** In a round-bottomed flask provided with nitrogen atmosphere and magnetic stirring was placed 6.0 g (0.02 mol) of *N*-protected phenylalanine. The amino acid was disolved in 20 mL of CH_3_CN and the flask was placed in an ice bath at 0 °C before the addition of 4.8 mL (0.044 mol, 2.2 equiv.) of *N*-methyl morpholine and 14.28 mL (0.024 mol, 1.2 equiv.) of a 1.68 M solution of propylphosphonic anhydride (T3P). The reaction mixture was stirred at 0 °C before the addition of additional 2.4 mL (0.022 mol, 1.1 equiv.) of *N*-methyl morpholine and 2.52 g (0.02 mol, 1 equiv.) of de HCl salt of glycine methyl ester, previously dissolved in 20 mL of CH_3_CN. The reaction mixture was allowed to reach ambient temperature and stirred for 24 additional hours. After this time, the solvent was evaporated and the crude was redisolved in 300 mL of EtOAc and washed with 1N HCl (2 × 150 mL) and saturated solution of sodium and potassium tartrate (1 × 100 mL). The organic extracts were dried with Na_2_SO_4_ and concentrated under vacuum. The product was crystallized from EtOAc:hexane (75:25) affording 5.8 g (78% yield) of the desired product as a white solid. Experimental properties in agreement with reported in literature [73]. Experimental mp 118–119 °C. ${\left[\alpha \right]}_{D}^{25}$ = +0.826 (c = 0.363, CHCl_3_). ^1^H-NMR (400 MHz, DMSO-*d_6_*, 120 °C) (ppm): 2.89 (dd, *J*_1_ = 9.2, *J*_2_ = 14.0 Hz, 1H), 3.11 (dd, *J*_1_ = 4.6, *J*_2_ = 14.0 Hz 1H), 3.67 (s, 3H), 3.89 (m, 2H), 4.38 (m, 1H), 5.01 (m, 2H), 6.79 (a, 1H), 7.19–7.36 (m, 10H), 7.94 (a, 1H); ^13^C-NMR (DMSO-*d_6_* / 100.52 MHz): δ 38.3, 41.4, 51.9, 56.6, 66.1, 126.6, 127.8, 128.0, 128.4, 128.6, 129.6, 137.6, 138.3, 156.0, 170.3, 172.1; HRESI-MS: *m/z* = 371.1598 [M + H]^+^; calculated for C_20_H_23_N_2_O_5_ 371.1601.

*((Benzyloxy)carbonyl)-l-phenylalanylglycine* (**22**). In a round-bottomed flask provided with magnetic stirring was placed 600 mg (1.6 mmol) of esther 21 and dissolved in 6 mL of THF. The flask was placed in an ice bath at 0 °C before the addition of 134.5 (3.2 mmol, 2 equiv.) of LiOH monohydrate in 2 mL of water. The reaction mixture was stirred at 0 °C and allowed to reach ambient temperature. After 24 h, the solvent was evaporated under vacuum and the residue was acidulated with conc. HCl to pH = 2 and then extracted with CH_2_Cl_2_. The organic extracts were dried with Na_2_SO_4_ and concentrated under vacuum. The product was crystallized from EtOAc:hexane affording 134 mg (23 % yield) of 22 as a white solid. Experimental properties in agreement with those reported in the literature [74], mp 128-130 °C. ${\left[\alpha \right]}_{D}^{25}$ = **−**10.59 (c = 0.34, AcOH). ^1^H-NMR (400 MHz, DMSO-*d_6_*, 120 °C) (ppm): 2.87 (dd, *J*_1_ = 9.2, *J*_2_ = 14.0 Hz, 1H), 3.12 (dd, *J*_1_ = 4.8, *J*_2_ = 9.2 Hz 1H), 3.66 (s, 2H), 4.33 (m, 1H), 5.00 (m, 2H), 6.85 (a, 1H), 7.16–7.36 (m, 10H), 7.57 (a, 1H); ^13^C-NMR (DMSO-*d_6_*, 100.52 MHz): δ 38.3, 42.8, 56.8, 66.0, 126.5, 127.7, 127.9, 128.4, 128.6, 129.6, 137.6, 138.6, 156.0, 170.3, 171.3; HRESI-MS: *m/z* = 357.1452 [M + H]^+^; calculated for C_19_H_21_N_2_O_5_, 357.1444. 

*l-Phenylalanylglycine* (**A**)**.** In a round bottomed flask provided with magnetic stirrer and H_2_ atmosphere was placed 86 mg of compound **22** with 8.6 mg of Pd/C 10% *w*/*w*. Cautiously, 2 mL of methanol was added and the reaction was stirred at ambient temperature for 24 h. Finally, the mixture was filtered on Celite and the solid washed with conc. NH_4_OH affording 56 mg (quantitative yield) of the desired product as a white solid. Experimental properties were in agreement with those reported in the literature [75], mp 218–220 °C (decomposes). ${\left[\alpha \right]}_{D}^{25}$ = −5.59 (c = 0.34, AcOH). ^1^H-NMR (500 MHz, D_2_O) (ppm): 2.84 (dd, *J*_1_ = 7.1, *J*_2_ = 13.6 Hz, 1H), 2.92 (dd, *J*_1_ = 6.7, *J*_2_ = 13.6 Hz, 1H), 3.42 (d, *J* = 17.3 Hz, 1H), 3.63 (d, *J* = 17.5 Hz,1H), 3.74 (dd, *J*_1_ = 6.9, *J*_2_ = 7.1 Hz 1H), 7.10–7.25 (m, 5H); ^13^C-NMR (D_2_O, 125.76 MHz): δ 38.9, 43.2, 55.6, 127.4, 128.7, 128.9, 129.4, 135.9, 173.4, 176.3; HRESI-MS: *m/z* = 223.1077 [M + H]^+^; calculated for C_11_H_14_N_2_O_3_, 223.1077.

*General method for addition of aldehydes to N-arylmaleimides:* 15.6 mg (0.05 mmol) of α,β-dipeptide **2**, 2 mg (0.05 mmol) of NaOH, 0.5 mmol (1 equiv.) of the corresponding maleimide and 2.75 mmol (5.5 equiv.) of the aldehyde were placed in a flask equipped with a magnetic stirrer. The reaction mixture was stirred for up to 24 h at ambient temperature, until thin layer chromatography (TLC) showed that the reaction was complete. The product was purified by flash column chromatography with a mixture of hexanes/EtOAc (7:3) as eluent. The absolute configuration of the products was assigned by comparison with the available literature [45,55,56,57,76,77]. The enantiomeric ratio was determined by chiral HPLC. NMR spectra and chromatograms can be found in Supplementary Materials.

*General method for the addition of isobutyraldehyde to nitroolefins:* 0.05 mmol of dipeptide **4** or **6**, 3.8 mg (0.05 mmol) of (thio)urea, 6.1 mg (0.05 mmol) of DMAP and 0.25 mL (2.75 mmol, 5.5 equiv.) of isobutyraldehyde were placed in a flask equipped with a magnetic stirrer. The resulting mixture was stirred for 5 min before the addition of 0.5 mmol (1 equiv.) of the corresponding nitroolefin. The reaction mixture was stirred for up to 24 h at ambient temperature until TLC showed that the reaction was complete. The product was purified by flash column chromatography with a mixture of hexanes/EtOAc (9:1) as eluent. The enantiomeric ratio was determined by chiral HPLC. The absolute configuration of the products was assigned in accordance with the literature [58,59,76,77].

## 5. Conclusions

Six novel α,β-dipeptides were evaluated as organocatalysts in the Michael addition reaction of various aldehydes to different substrates. With *N*-arylmaleimides or nitroolefins as electrophiles, the dipeptide alone was not able to promote the reaction, whereas with base NaOH as additive the asymmetric Michael addition to maleimides proceeds. Similarly, a 1:1 mixture of DMAP and urea or thiourea promotes the Michael addition reaction to β-nitrostyrene. The effective use of additives reported in this work constitutes a clear example of how catalytic activity depends markedly on non-covalent interactions induced by the additive which are apparently more efficient under solvent-free reaction conditions. In particular, the sodium salt of the dipeptidic catalyst as well as a putative supramolecular cluster consisting of dipeptide, DMAP and (thio)urea appear to play a key role in the stereoselective Michael addition reactions presented here.

## Figures and Tables

**Table 1:**
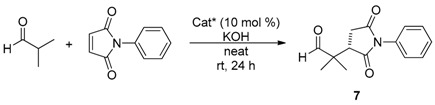
Michael addition reaction of isobutyraldehyde to *N*-phenylmaleimide organocatalyzed by α,β-dipeptides **1**–**6**.

Essay ^a^	Cat*	% Yield ^b^	er ^c^
1	**1**	79	76:24
2	**2**	64	88:12
3	**3**	77	77:23
4	**4**	65	79:21
5	**5**	75	88:12
6	**6**	74	85:15

^a^ Reaction conditions: aldehyde (5.5 mmol), maleimide (0.5 mmol), cat* 10 mol % (0.05 mmol), and KOH, 10 mol % (0.05 mmol). ^b^ Isolated yield. ^c^ Determined by chiral HPLC. rt, room temperature; er, enantiomeric ratio; Cat*, chiral catalyst.

**Table 2:**
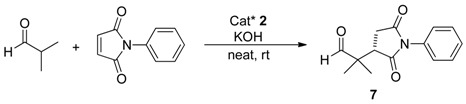
Michael addition reaction of isobutyraldehyde to *N*-phenylmaleimide with different amounts of catalyst.

Essay ^a^	Cat* (mol %)	% Yield ^b^	er ^c^
1	1	66	63:37
2	2	71	62:38
3	5	74	73:27
**4**	**10**	**73**	**86**:**14**
5	15	72	67:33
6	20	76	65:35
7	25	56	63:37

^a^ Reaction conditions: aldehyde (5.5 mmol), maleimide (0.5 mmol), KOH was used in the same amount as catalyst **2**. ^b^ Isolated yield. ^c^ Determined by chiral HPLC.

**Table 3:**
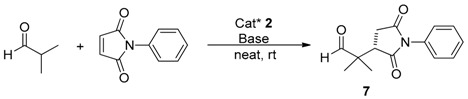
Michael addition reaction of isobutyraldehyde to *N*-phenylmaleimide organocatayzed by phenylalanine-β-alanine (Phe-β-Ala, **2**) in the presence of different bases.

Essay ^a^	Base	% Yield ^b^	er ^c^
1	------	n.r.	n.d.
2	LiOH	63	84:16
**3**	**NaOH**	**69**	**90**:**10**
4	NaHCO_3_	54	73:27
5	KHCO_3_	45	70:30
6	Na_2_CO_3_	57	65:35
7	K_2_CO_3_	31	68:32
8	Cs_2_CO_3_	26	58:42
9	NMM	66	55:45
10	Et_3_N	10	49:51
11	DMAP	traces	58:42

^a^ Reaction conditions: aldehyde (2.75 mmol), maleimide (0.5 mmol), KOH was used in the same amount as catalyst **2**. ^b^ Isolated yield. ^c^ Determined by chiral HPLC. NMM, *N*-methylmorpholine; DMAP, 4-dimethylaminopyridine.

**Table 4:**
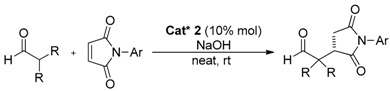
Michael addition reaction of aldehydes to different *N*-substituted maleimide substrates.

Essay ^a^	R	Ar	Product	% Yield ^b^	er ^c^
1	CH_3_	4-Cl-C_6_H_4_-	**9**	63	81:19
2	CH_3_	4-Br-C_6_H_4_-	**10**	39	83:17
3	CH_3_	2-MeO-C_6_H_4_-	**11**	traces	n.d.
5	CH_3_	3-Cl-C_6_H_4_-	**12**	70	69:31
6	–(CH_2_)_5_–	C_6_H_5_-	**13**	n.r.	n.d.
8	CH_2_CH_3_	C_6_H_5_-	**14**	47	74:26

^a^ Reaction conditions: aldehyde (2.75 mmol), *N*-substituted maleimide (0.5 mmol), **2** and KOH both 10 mol % (0.05 mmol). ^b^ Isolated yield. ^c^ Determined by chiral HPLC.

**Table 5:**
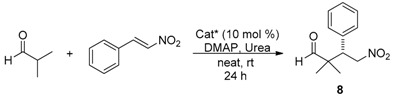
Michael addition reaction of isobutyraldehyde to *trans*-β-nitrostyrene organocatalyzed by α,β-dipeptides **1**–**6**.

Essay ^a^	Cat*	% Yield ^b^	er ^c^
1	**1**	59	74:26
2	**2**	72	82:18
3	**3**	70	77:23
**4**	**4**	**96**	**92**:**8**
5	**5**	13	88:12
**6**	**6**	**98**	**93**:**7**

^a^ Reaction conditions: aldehyde (5.5 mmol), *trans-*β-nitrostyrene (0.5 mmol), cat* (10 mol %), DMAP (10 mol %), urea (10 mol %). ^b^ Isolated yield. ^c^ Determined by chiral HPLC.

**Table 6:**
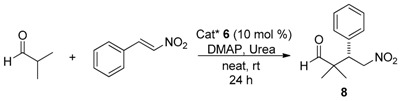
Michael addition reaction of isobutyraldehyde to *trans*-β-nitrostyrene with different amounts of catalyst.

Essay ^a^	Cat* (mol %)	% Yield ^b^	er ^c^
1	2	n.r.	n.d.
2	5	42	93:7
**3**	**10**	**98**	**93**:**7**
4	15	89	92:2
5	20	81	92:2

^a^ Reaction conditions: aldehyde (2.75 mmol), *trans*-β-nitrostyrene (0.5 mmol) DMAP and urea were used in the same amount as cat*: 10 mol % (0.05 mmol). ^b^ Isolated yield. ^c^ Determined by chiral HPLC.

**Table 7:**
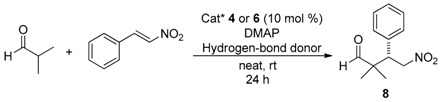
Michael addition reaction of isobutyraldehyde to *trans*-β-nitrostyrene organocatalyzed by dipeptides **4** and **6** in the presence of different hydrogen bond donors and at various temperatures.

Essay ^a^	Cat*	Hydrogen Bond Donor	Temp (°C)	% Yield ^b^	er ^c^
1	**4**	------	25	n.r.	n.d.
2	**4** + NaOH	------	25	n.r.	n.d.
3	**4**	Urea	25	96	90:10
4	**4**	Thiourea	25	79	91:9
5	**4**	Sulphamide	25	84	83:17
**6**	**6**	**Urea**	**25**	**98**	**93**:**7**
**7**	**6**	**Thiourea**	**25**	**98**	**92**:**8**
8	**6**	Sulphamide	25	91	93:7
9	**6**	Urea	2	72	94:6
10	**6**	Urea	−15	57	95:5

^a^ Reaction conditions: aldehyde (2.75 mmol), *trans*-β-nitrostyrene (0.5 mmol), additives (DMAP + hydrogen bond donor was used in 10 mol %). ^b^ Isolated yield. ^c^ Determined by chiral HPLC.

**Table 8:**
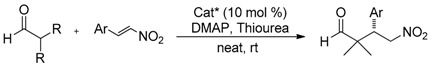
Michael addition reaction of aldehydes to different substrates.

Essay ^a^	Cat*	R	Ar	Product	% Yield ^b^	er ^c^
1	**4**	CH_3_	2-MeO-C_6_H_4_-	**15**	78	86:14
	**6**				77	87:13
2	**4**	CH_3_	2-Br-C_6_H_4_-	**16**	31	85:15
	**6**				29	83:13
3	**4**	CH_3_	4-MeO-C_6_H_4_-	**17**	52	91:9
	**6**				83	91:9
4	**4**	CH_3_	4-Me-C_6_H_4_-	**18**	95	91:9
	**6**				56	93:7
5	**4**	CH_3_	4-Cl-C_6_H_4_-	**19**	77	85:15
	**6**				45	91:9
6	**4**	CH_3_	4-F-C_6_H_4_-	**20**	69	90:10
	**6**				61	92:8

^a^ Reaction conditions: aldehyde (2.75 mmol), *trans*-β-nitrostyrene (0.5 mmol), additives (DMAP + thiourea were used in 10 mol %). ^b^ Isolated yield. ^c^ Determined by chiral HPLC.

## Supplementary Materials

The following are available online. References [45,57,59,76,77,78] are cited in the supplementary materials.
