# Supplementary material for: Asymmetric Michael Addition Organocatalyzed by α,β-Dipeptides under Solvent-Free Reaction Conditions
Source: Molecules. 2017 Aug 10;22(8):1328. doi: 10.3390/molecules22081328 (PMC6152315; doi:10.3390/molecules22081328)
Supplement: Supplementary file 1 [file molecules-22-01328-s001.pdf]

Asymmetric Michael Addition Organocatalyzed by α,β-Dipeptides Under Solvent-free Reaction Conditions

C. Gabriela Avila-Ortiz [1,], Lenin Díaz-Corona, Erika Jiménez-González,[1] Eusebio Juaristi [1,2,*]

[1] Departamento de Química, Centro de Investigación y de Estudios Avanzados, Instituto Politécnico Nacional, Avenida I. P. N. 2508, 07360-Ciudad de México, Mexico.

[2] El Colegio Nacional, Luis González Obregón 23, Centro Histórico, 06020-Ciudad de México, México.

***** Author to whom correspondence should be addressed; E-Mail: juaristi@relaq.mx; Tel.: +52-55-5747-3722; Fax: +52-55-5747-3897.

Supporting Information

Table of contents

1. Synthesis of L-phenylalanylglycine 2

2. Reaction Optimization Tables 3

3. Experimental Properties of the Michael Adducts 6

4. Bibliography 30

1. Synthesis of L-phenylalanylglycine

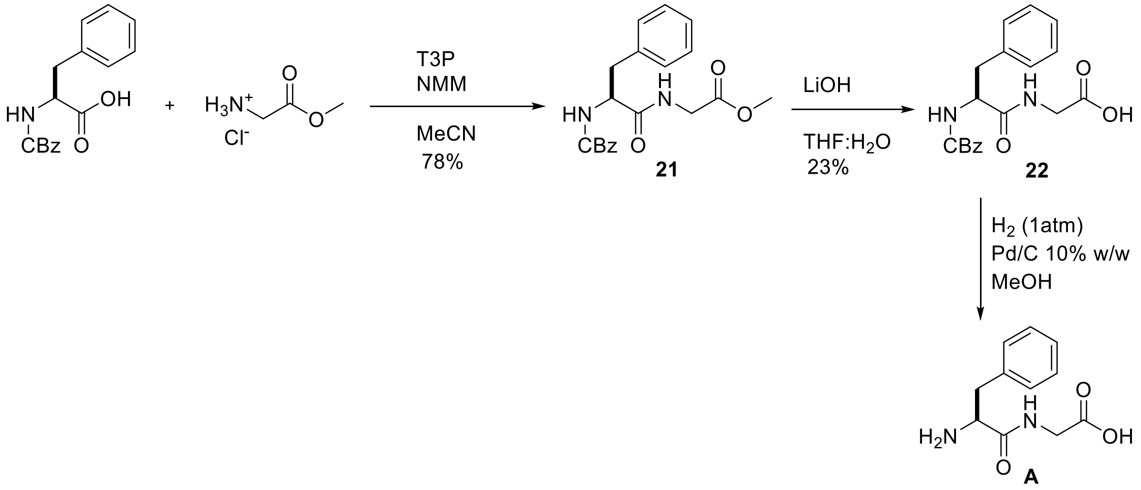

Scheme A: Synthetic route of L-phenylalanylglycine

2. Reaction Optimization Tables

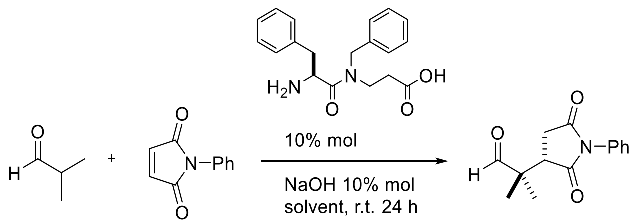

Table A. Effect of the solvent on the Michael addition reaction of isobutyraldehyde to *N*-phenylmaleimide.

Essay[a]	Solvent	Yield[b] [%]	er[c]
1	EtOH	n.r.	n.d.
2	Toluene	n.r.	n.d.
3	H_2O	n.r.	n.d.
4	CH_2Cl_2	77	88 : 12
5	Brine	n.r.	n.d.
6	THF	n.r.	n.d.
7	DMSO	n.r.	n.d.
8	MeOH	n.r.	n.d.
9	CH_3CN	n.r.	n.d.

[a] Reaction conditions: aldehyde (5.5 mmol), maleimide (0.5 mmol), NaOH was used in the same amount as **2**, and 0.5 mL of the indicated solvent. [b] Isolated yield. [c] Determined by chiral HPLC.

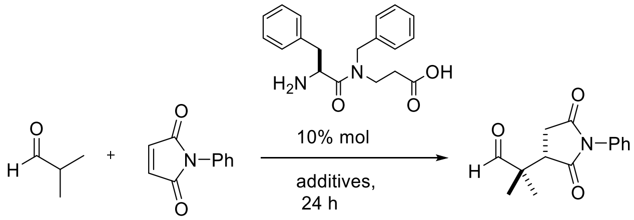

Table B: Effect of additives and temperature on the Michael addition reaction of isobutyraldehyde to *N*-phenylmaleimide.

Essay[a]	H--Donor	Base	Temperature [° C]	Yield[b] [%]	er[c]
1	Urea	NaOH	25	80	68 : 32
2	Urea	DMAP	25	40	71 : 29
3	Thiourea	NaOH	25	75	73 : 27
4	Thiourea	DMAP	25	43	n. d.
5	Sulphamide	NaOH	25	67	75 : 25
6	Sulphamide	DMAP	25	16	n. d.
7	Urea	NaOH	2	80	65 : 35
8	Thiourea	NaOH	2	81	70 : 30
9	Sulphamide	NaOH	2	82	66 : 34
10	------	NaOH	2	79	86 : 14

[a] Reaction conditions: aldehyde (2.75 mmol), maleimide (0.5 mmol), additives were used in the same amount as catalyst **2**. [b] Isolated yield. [c] Determined by chiral HPLC

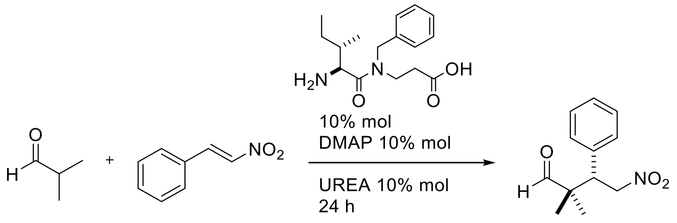

Table C. Effect of the solvent on the Michael addition reaction of isobutyraldehyde to *trans*-β-nitrostyrene.

Essay[a]	Solvent	Yield[b] [%]	er[c]
1	DMSO	n.r.	n.d.
2	H_2O	81	93 : 7
3	DMF	n.r.	n.d.
4	MeOH	n.r.	n.d.
5	CH_3CN	n.r.	n.d.
6	CH_2Cl_2	64	94 : 6
7	iPrOH	n.r.	n.d.
8	Brine	n.r.	n.d.
9	THF	n.r.	n.d.

[a] Reaction conditions: aldehyde (2.75 mmol), *trans*-β-nitrostyrene (0.5 mmol), Additives (DMAP + Hydrogen-bond donor were used in 10% mol) and 0.5 mL of the solvent. [b] Isolated yield. [c] Determined by chiral HPLC.

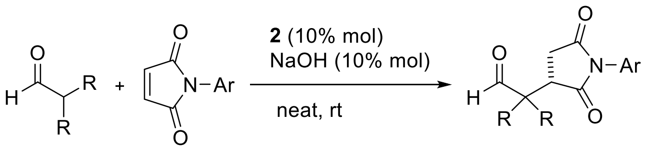

Table D. Michael Addition Reaction of Aldehydes to Additional Substrates.

Essay[a]	R	Ar	Product	Yield[b]	er[c]
1	CH_3	2-NO_2-C_4H_4-	**23**	n.r.	n.d.
2	-$(CH_2)_4$-	C_6H_5-	**24**	n.r.	n.d.

[a] Reaction conditions: aldehyde (2.75 mmol), *N*-substituted maleimide (0.5 mmol), cat* and KOH both 10% mol (0.05 mmol). [b] Isolated yield. [c] Determined by chiral HPLC.

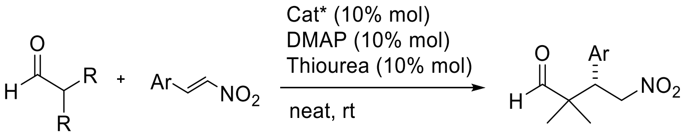

Table E. Michael Addition Reaction of Aldehydes to Different Substrates.

Essay[a]	Cat*	R	Ar	Product	Yield[b]	er[c]
7	**4**	-$(CH_2)_4$-	C_6H_5-	**25**	n.r.	n.d.
	**6**				n.r.	n.d.
8	**4**	-$(CH_2)_5$-	C_6H_5-	**26**	n.r.	n.d.
	**6**				n.r.	n.d.
9	**4**	CH_2CH_3	C_6H_5-	**27**	n.r.	n.d.
	**6**				n.r.	n.d.

[a] Reaction conditions: aldehyde (2.75 mmol), *trans*-β-nitrostyrene (0.5 mmol), Additives (DMAP + thiourea were used in 10% mol). [b] Isolated yield. [c] Determined by chiral HPLC.

3. Experimental Properties of the Michael Adducts

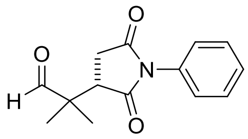

(*S*)-2-(2,5-Dioxo-1-phenylpyrrolidin-3-yl)-2-methylpropanal, 7

Optical rotation were in agreement with those reported in the literature.[1,2] NMR spectra and HPLC chromatograms were in agreement with those reported for the enantiomer in the literature.[3]

^{1}H NMR (270 MHz, $CDCl_3$) (ppm): 1.25 (s, 3H), 1.30 (s, 3H), 2.59 (dd, J_1 = 5.5, J_2 = 18.1 Hz, 1H), 2.77 (dd, J_1= 9.5, J_2 = 18.1 Hz, 1H), 3.12 (dd, J_1 = 5.4, J_2 = 9.5 Hz, 1H), 7.23-7.48 (m, 5H), 9.48 (s, 1H).

^{13}C NMR (67.93 MHz, $CDCl_3$) (ppm): 19.6, 20.4, 31.9, 45.06, 48.6, 126.6, 128.8, 129.3, 131.8, 174.9, 177.0, 202.8.

$[\alpha]_D^{25°C}$ = –1.1 (c = 0.333, $CHCl_3$).

HPLC (Chiralcel OD-H, hexane/i-PrOH 80/20, 1 mL/min, λ = 210 nm): t_{major} = 17 min, t_{minor} =22 min.

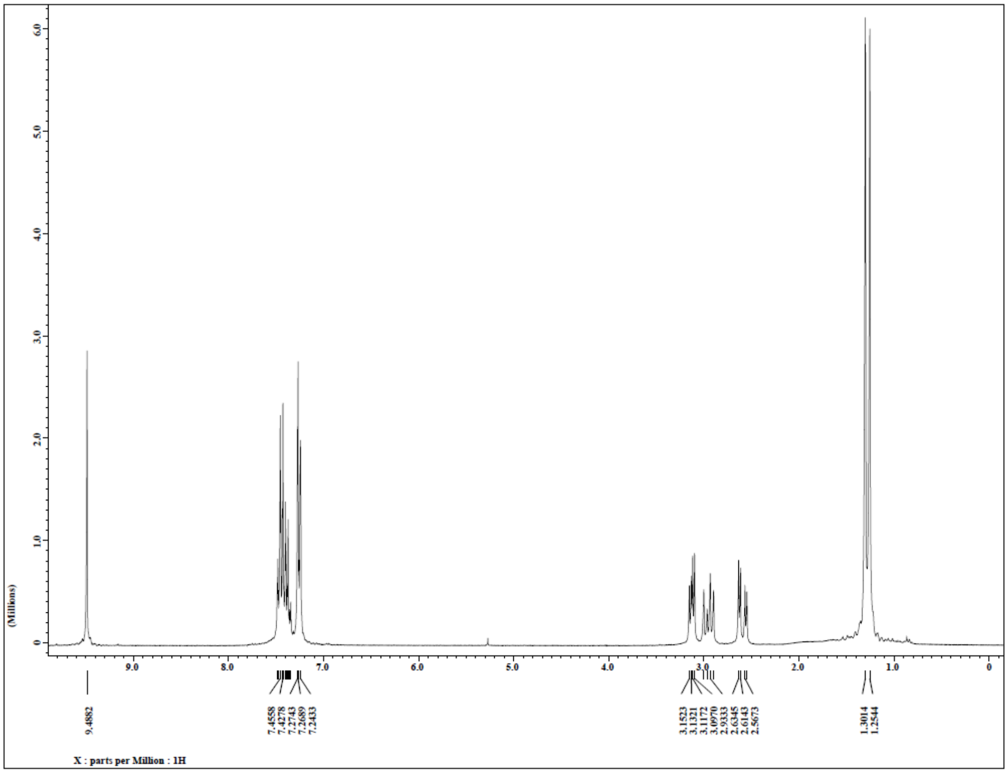

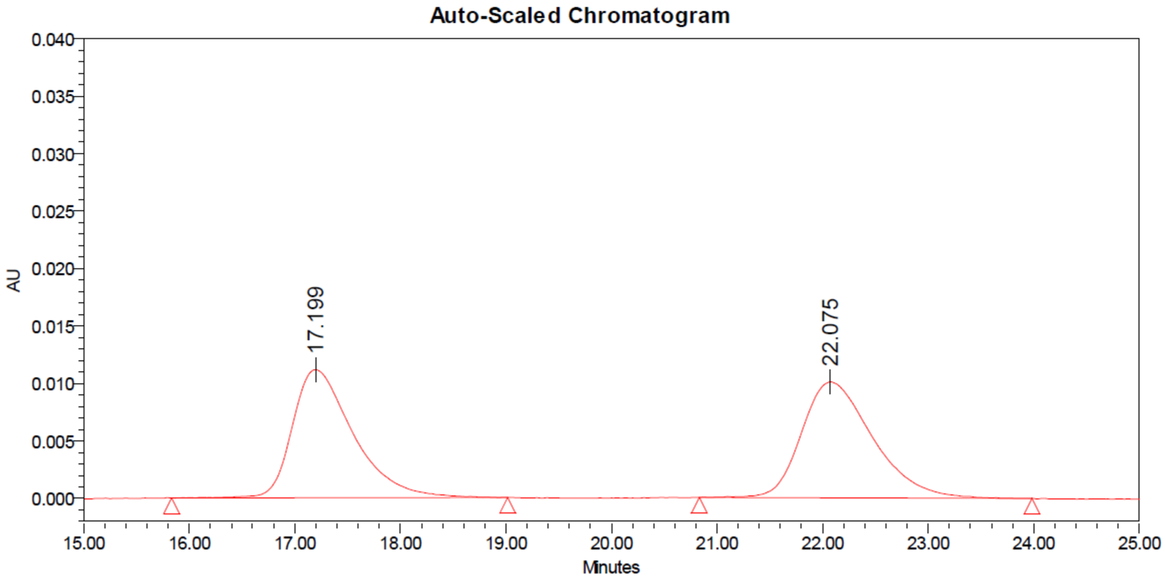

Peak Results

	RT	Area	Height	% Area
1	17.199	446710	11149	48.56
2	22.075	473221	10096	51.44

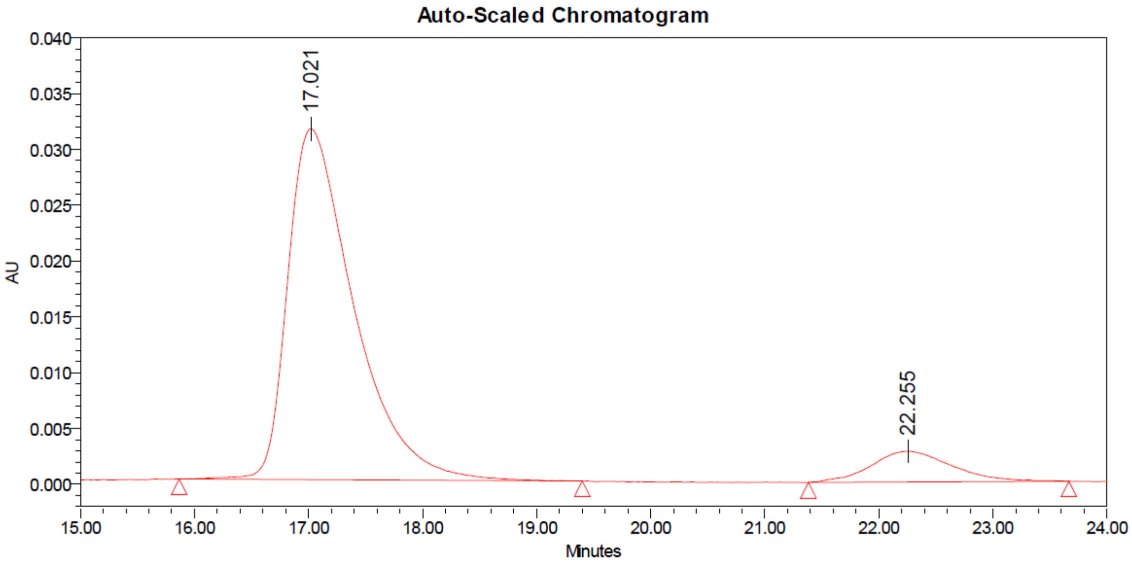

Peak Results

	RT	Area	Height	% Area
1	17.021	1266540	31437	90.36
2	22.255	135076	2753	9.64

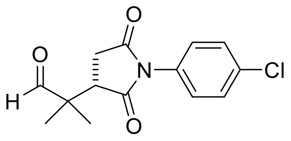

(*S*)-2-(1-(4-Chlorophenyl)-2,5-dioxopyrrolidin-3-yl)-2-methylpropanal, 9

Optical rotation was in agreement with that previously reported in the literature.[2] NMR and HPLC in agreement with those reported in the literature for the enantiomeric compound.[3]

^{1}H NMR (500 MHz, $CDCl_3$) (ppm): 1.25 (s, 3H), 1.32 (s, 3H), 2.59 (dd, J_1 = 5.6, J_2 = 18.4 Hz, 1H), 2.94 (dd, J_1= 9.6, J_2 = 18.3 Hz, 1H), 3.08 (dd, J_1 = 5.6, J_2 = 9.5 Hz, 1H), 7.23 (d, $J_{A\text{-}B}$ = 8.8 Hz, 2H), 7.32 (d, J_{AB} = 8.8, 2H), 9.46 (s, 1H).

^{13}C NMR (125.76 MHz, $CDCl_3$) (ppm): 19.9, 20.6, 32.0, 45.0, 48.8, 127.9, 129.5, 130.4, 134.6, 174.6, 176.8, 202.9.

$[\alpha]_D^{25°C}$ = –1.5 (c = 0.333, CH_2Cl_2).

HPLC (Chiralcel OD-H, hexane/i-PrOH 75/25, 0.6 mL/min, λ = 210 nm): t_{major} = 27 min, t_{minor} = 44 min.

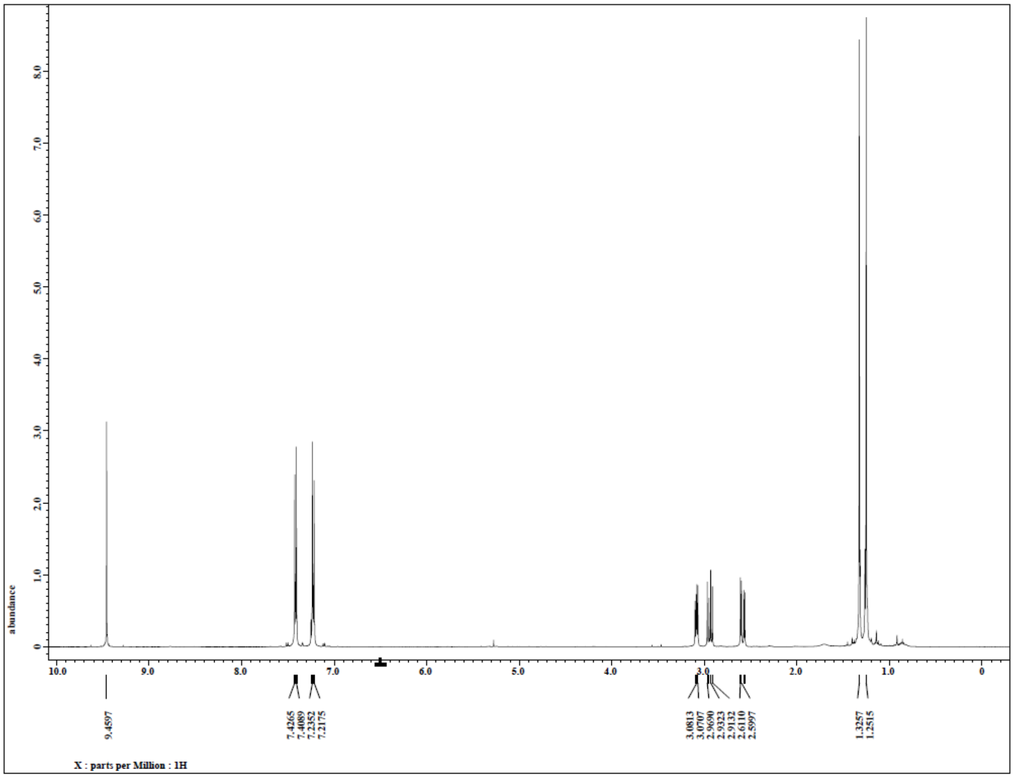

Chromatogram

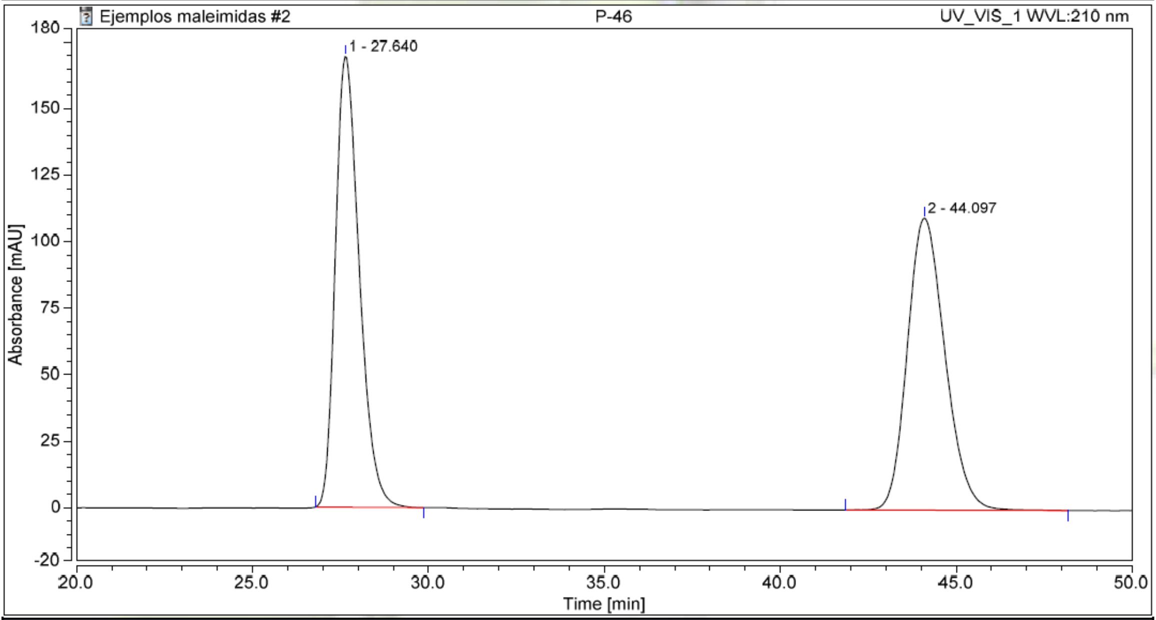

Integration Results

No.	Peak Name	Retention Time min	Area mAU*min	Height mAU	Relative Area %	Relative Height %	Amount n.a.
1		27.640	133.746	169.707	49.60	60.71	n.a.
2		44.097	135.911	109.829	50.40	39.29	n.a.
**Total:**			**269.657**	**279.536**	**100.00**	**100.00**	

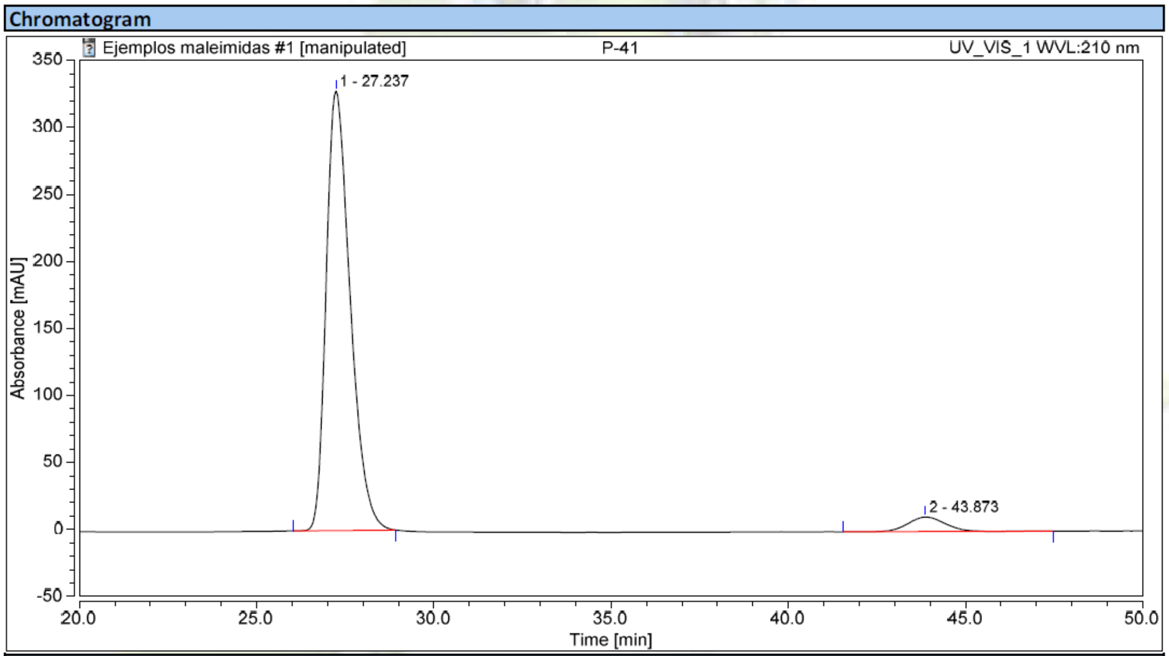

Integration Results

No.	Peak Name	Retention Time min	Area mAU*min	Height mAU	Relative Area %	Relative Height %	Amount n.a.
1		27.237	257.376	328.131	95.16	96.81	n.a.
2		43.873	13.090	10.818	4.84	3.19	n.a.
**Total:**			**270.467**	**338.949**	**100.00**	**100.00**	

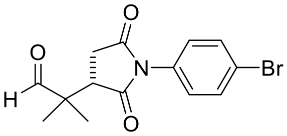

(*S*)-2-(1-(4-Bromophenyl)-2,5-dioxopyrrolidin-3-yl)-2-methylpropanal, 10.

Optical rotation was in agreement with that previously reported.[1] NMR spectra and HPLC in agreement with those reported in the literature for the enantiomeric compound.[3]

^{1}H NMR (500 MHz, $CDCl_3$) (ppm): 1.27 (s, 3H), 1.34 (s, 3H), 2.59 (dd, J_1 = 5.6, J_2 = 18.4 Hz, 1H), 2.95 (dd, J_1= 9.5, J_2 = 18.3 Hz, 1H), 3.09 (dd, J_1 = 5.6, J_2 = 9.5 Hz, 1H), 7.17 (d, $J_{A\text{-}B}$ = 8.6 Hz, 2H), 7.58 (d, J_{AB} = 8.6, 2H), 9.47 (s, 1H).

^{13}C NMR (125.76 MHz, $CDCl_3$) (ppm): 20.0, 20.6, 32.0, 45.1, 48.8, 122.6, 128.1, 130.9, 132.4, 174.5, 176.6, 202.8.

$[\alpha]_D^{25°C}$ = –1.2 (c = 0.3, $CHCl_3$).

HPLC (Chiralcel OD-H, hexane/i-PrOH 75/25, 0.6 mL/min, λ = 210 nm): t_{major} = 28.5 min, t_{minor} = 47.6 min.

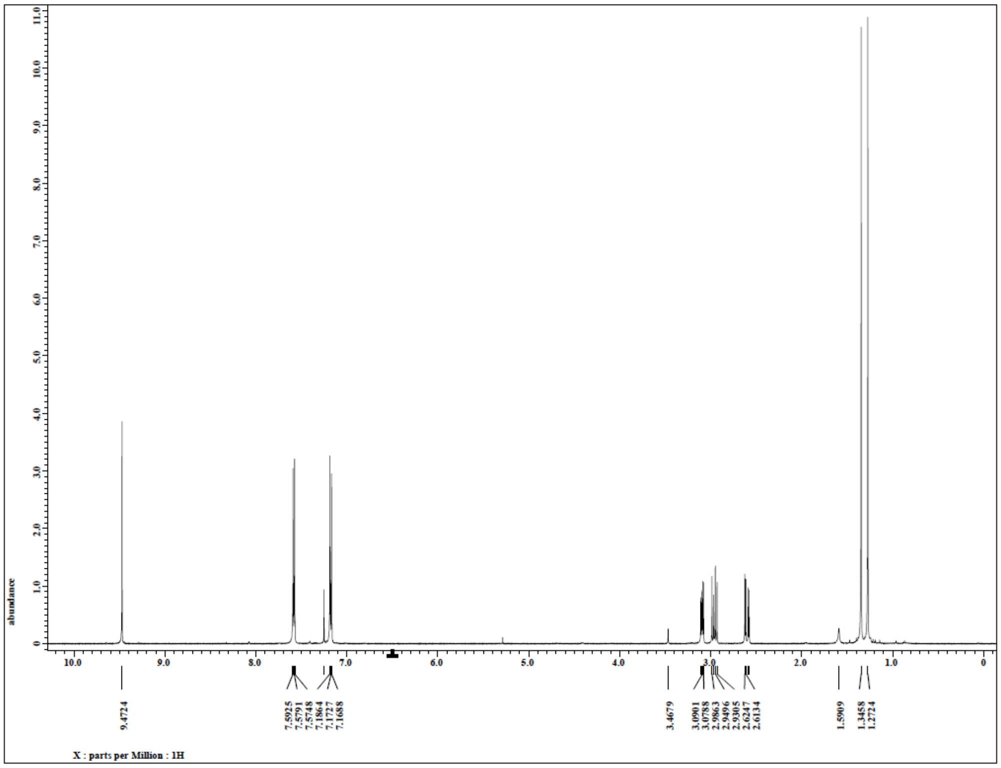

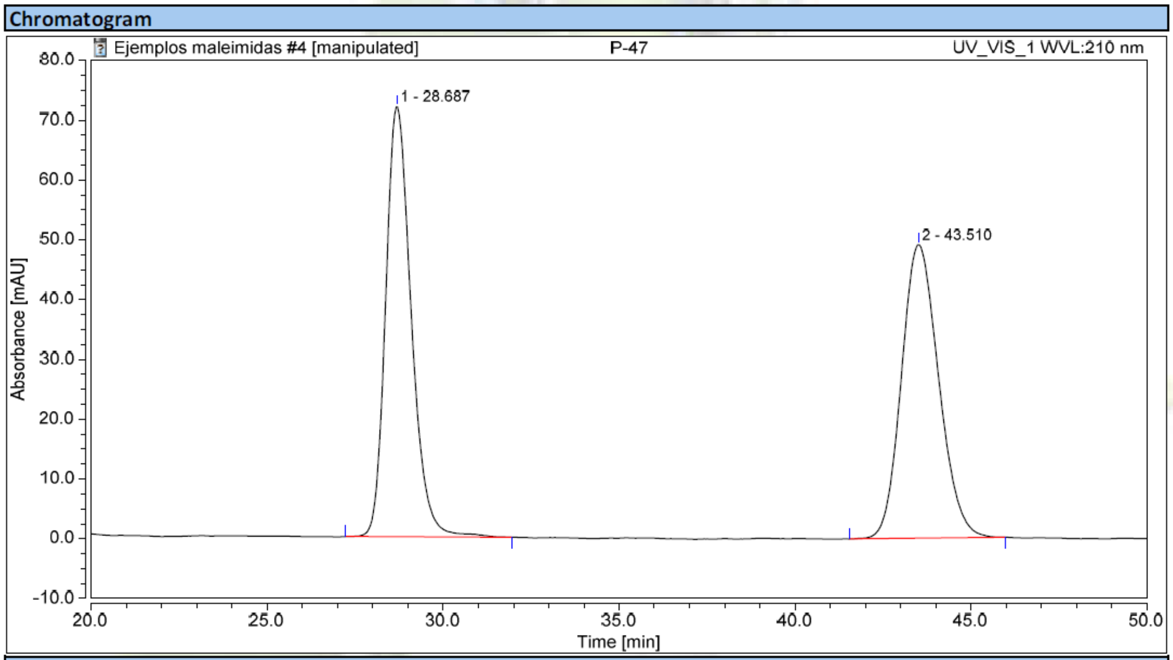

Integration Results

No.	Peak Name	Retention Time min	Area mAU*min	Height mAU	Relative Area %	Relative Height %	Amount n.a.
1		28.687	60.601	72.071	50.20	59.46	n.a.
2		43.510	60.118	49.136	49.80	40.54	n.a.
**Total:**			**120.719**	**121.207**	**100.00**	**100.00**	

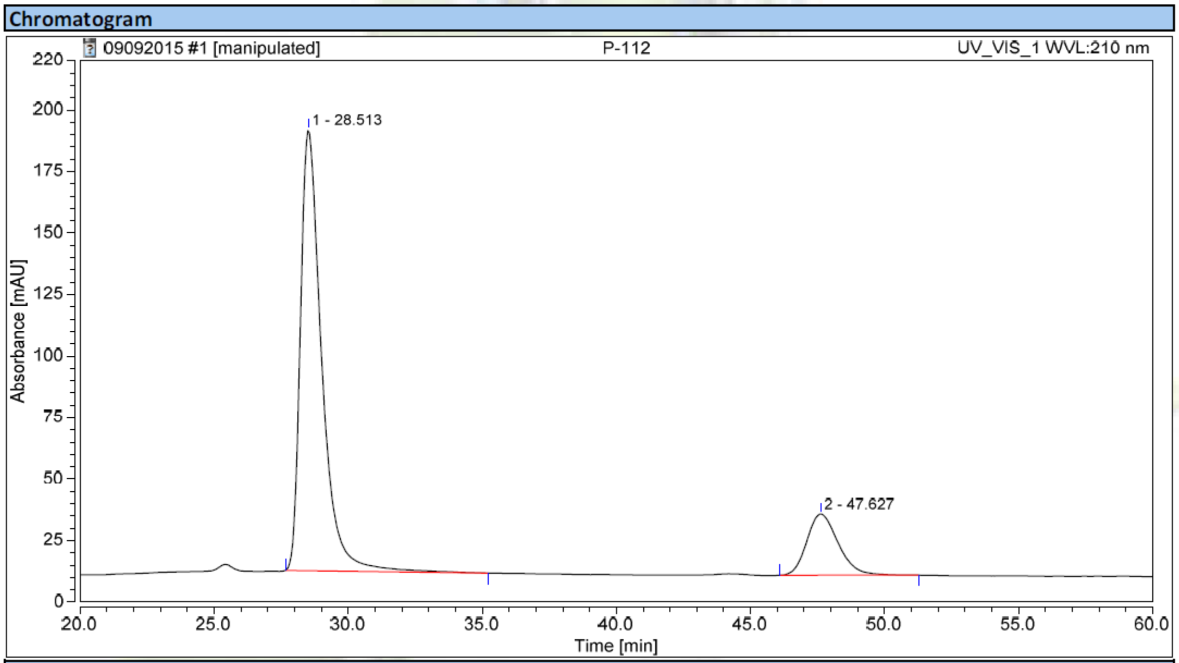

Integration Results

No.	Peak Name	Retention Time min	Area mAU*min	Height mAU	Relative Area %	Relative Height %	Amount n.a.
1		28.513	164.938	179.075	83.02	87.80	n.a.
2		47.627	33.731	24.882	16.98	12.20	n.a.
**Total:**			**198.669**	**203.958**	**100.00**	**100.00**	

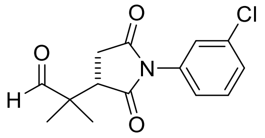

(*S*)-2-(1-(3-Chlorophenyl)-2,5-dioxopyrrolidin-3-yl)-2-methylpropanal, 12

NMR spectra and HPLC were in agreement with those reported in the literature for the enantiomeric compound.[3]

^{1}H NMR (500 MHz, $CDCl_3$) (ppm): 1.28 (s, 3H), 1.36 (s, 3H), 2.61 (dd, J_1 = 5.6, J_2 = 18.3 Hz, 1H), 2.97 (dd, J_1= 9.5, J_2 = 18.3 Hz, 1H), 3.10 (dd, J_1 = 5.6, J_2 = 9.7 Hz, 1H), 7.10-7.42 (m, 4H), 9.48 (s, 1H).

^{13}C NMR (125.76 MHz, $CDCl_3$) (ppm): 20.0, 20.6, 32.0, 45.0, 48.8, 124.8, 126.9, 129.0, 130.2, 132.9, 134.8, 174.4, 176.6, 202.8.

$[\alpha]_D^{25°C}$ = –5.0 (c = 0.3, $CHCl_3$).

HPLC (Chiralcel OD-H, hexane/i-PrOH 75/25, 0.9 mL/min, λ = 210 nm): t_{major} = 20.8 min, t_{minor} = 25.2 min.

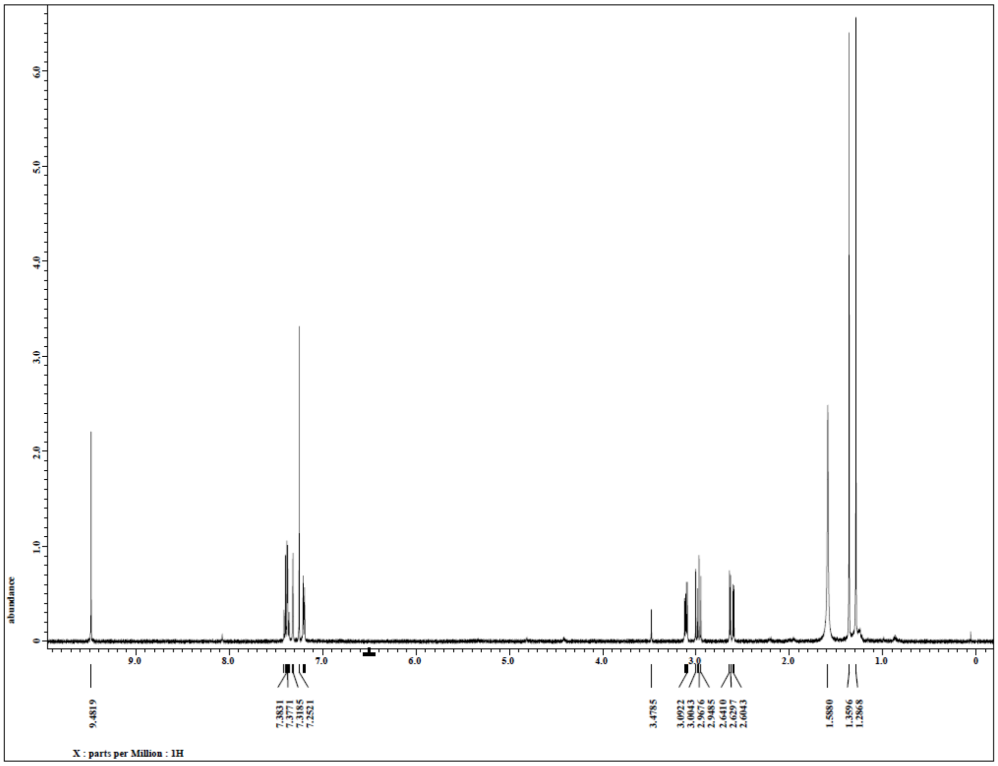

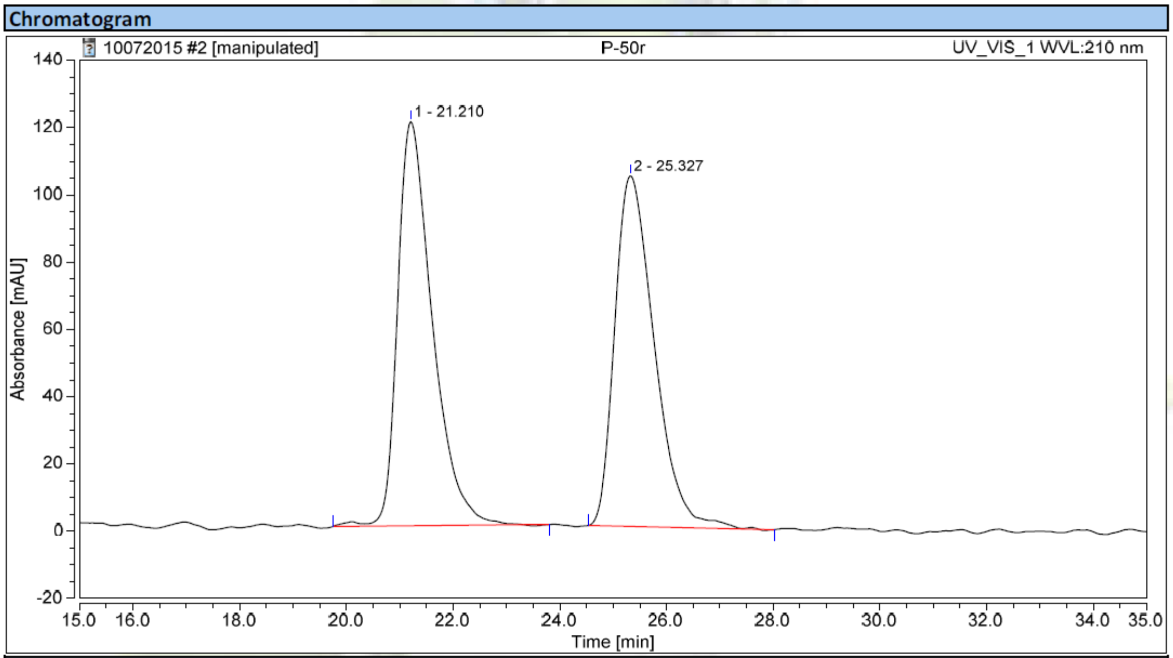

Integration Results

No.	Peak Name	Retention Time min	Area mAU*min	Height mAU	Relative Area %	Relative Height %	Amount n.a.
1		21.210	90.742	120.191	50.44	53.53	n.a.
2		25.327	89.157	104.326	49.56	46.47	n.a.
**Total:**			**179.899**	**224.517**	**100.00**	**100.00**	

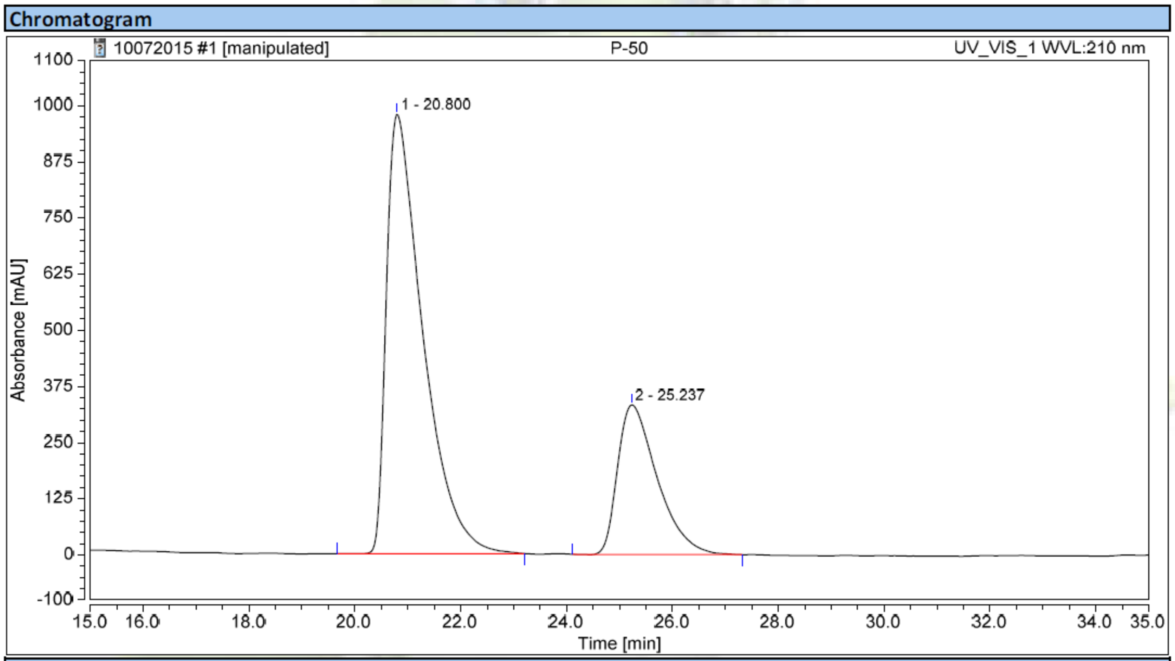

Integration Results

No.	Peak Name	Retention Time min	Area mAU*min	Height mAU	Relative Area %	Relative Height %	Amount n.a.
1		20.800	780.048	978.613	73.02	74.59	n.a.
2		25.237	288.238	333.368	26.98	25.41	n.a.
**Total:**			**1068.287**	**1311.980**	**100.00**	**100.00**	

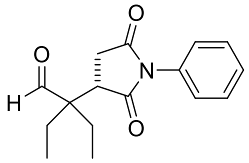

(*S*)-2-(2,5-Dioxo-1-phenylpyrrolidin-3-yl)-2-ethylbutanal, 14

Optical rotation was in agreement with that reported in the literature.[1] NMR spectra and HPLC in agreement with those reported in the literature for the enantiomeric compound.[3]

^{1}H NMR (500 MHz, $CDCl_3$) (ppm): 0.94 (t, J = 4.5 Hz. 3H), 0.98 (t, J = 4.5, 3H), 1.62-2.20 (m, 4H), 2.64 (dd, J_1 = 5.8, J_2 = 18.4 Hz, 1H), 2.93 (dd, J_1 = 9.5, J_2 = 18.3 Hz, 1H), 3.22 (dd, J_1 = 5.8, J_2 = 9.5 Hz, 1H), 7.23-7.45 (m, 5H), 9.59 (s, 1H).

^{13}C NMR (125.76 MHz, $CDCl_3$) (ppm): 8.14, 8.23, 23.3, 24.4, 31.8, 41.9, 54.6, 126.6, 128.7, 129.2, 132.0, 175.1, 177.4, 204.2.

$[\alpha]_D^{25°C}$ = –8.0 (c = 0.3, CH_2Cl_2).

HPLC (Chiralcel OD-H, hexane/i-PrOH 75/25, 0.5 mL/min, λ = 210 nm): t_{major} = 30.0 min, t_{minor} = 52.3 min.

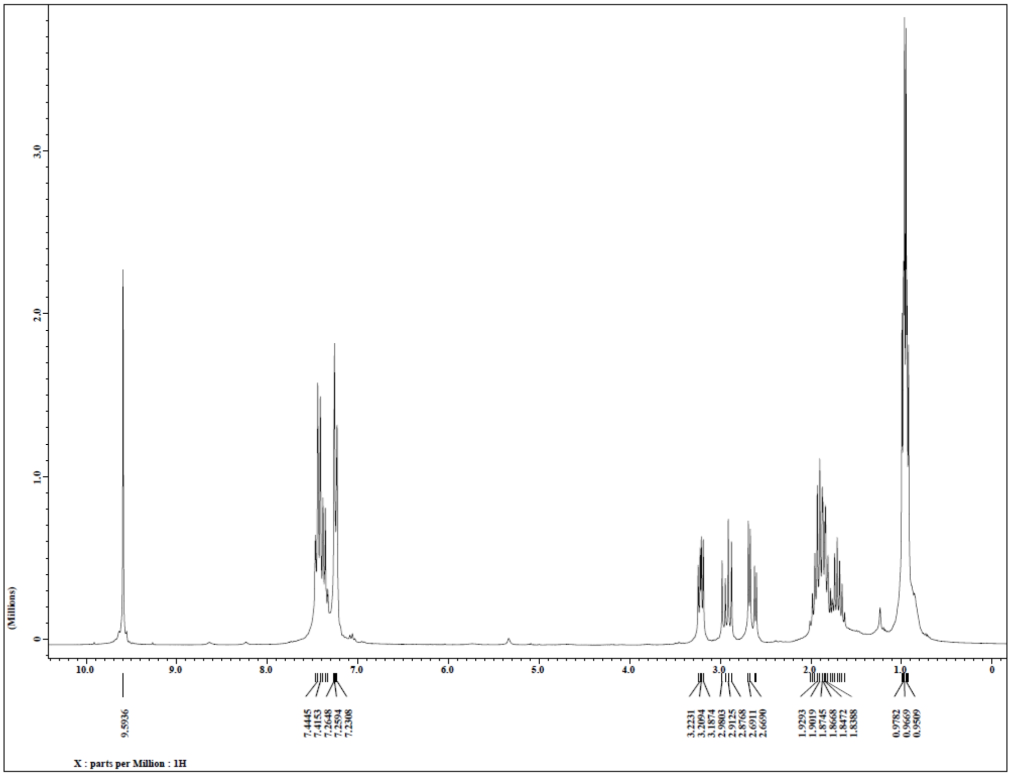

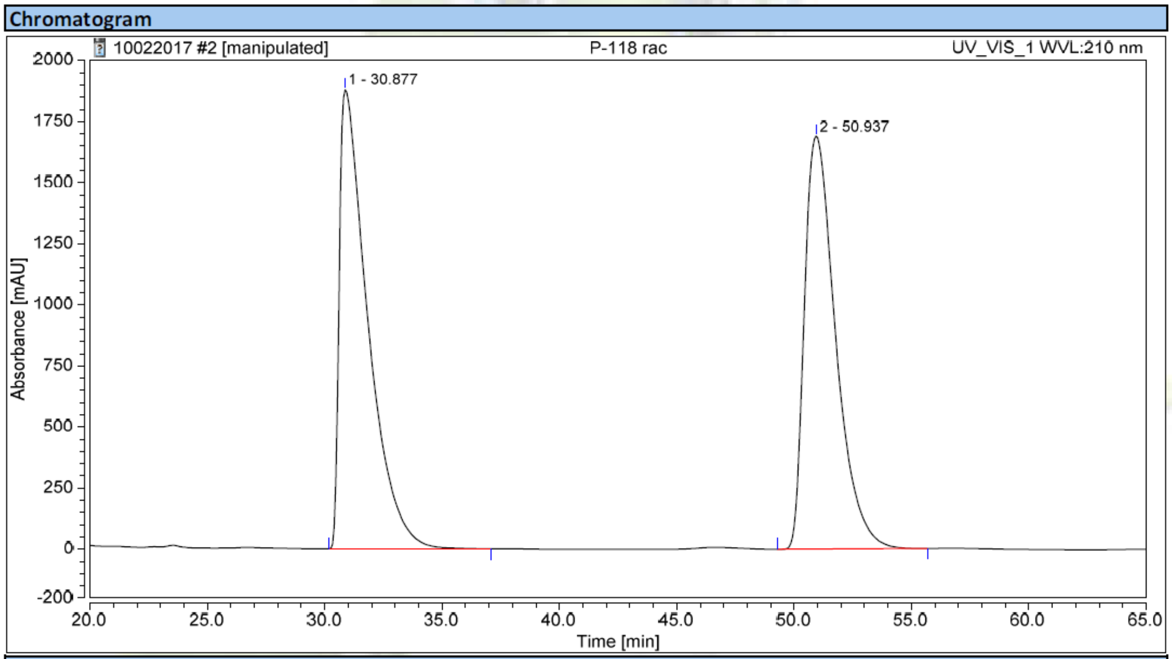

Integration Results

No.	Peak Name	Retention Time min	Area mAU*min	Height mAU	Relative Area %	Relative Height %	Amount n.a.
1		30.877	2630.756	1878.433	50.20	52.64	n.a.
2		50.937	2609.411	1690.319	49.80	47.36	n.a.
**Total:**			**5240.167**	**3568.752**	**100.00**	**100.00**	

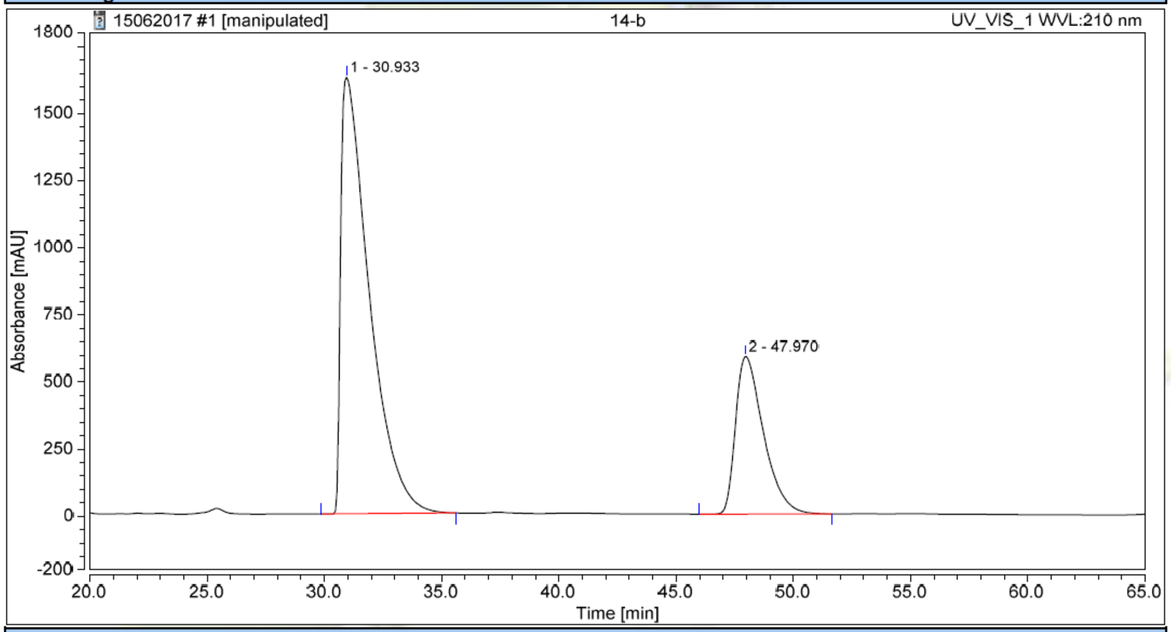

Integration Results

No.	Peak Name	Retention Time min	Area mAU*min	Height mAU	Relative Area %	Relative Height %	Amount n.a.
1		30.933	2287.719	1627.038	73.84	73.44	n.a.
2		47.970	810.558	588.282	26.16	26.56	n.a.
**Total:**			**3098.277**	**2215.320**	**100.00**	**100.00**	

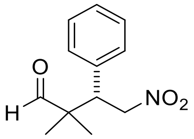

(*S*)-2,2-Dimethyl-4-nitro-3-phenylbutanal, 8

Optical rotation was in agreement with that previously reported.[4,5] NMR spectra and HPLC in agreement with those reported in the literature.[6]

^{1}H NMR (500 MHz, $CDCl_3$) (ppm): 0.99 (s, 3H), 1.12 (s, 3H), 3.78 (dd, J_1 = 3.9, J_2 = 11.3 Hz, 1H), 4.68 (dd, J_1= 3.9, J_2 = 13.1 Hz, 1H), 4.85 (dd, J_1 = 11.3, J_2 = 13 Hz, 1H), 6.86-6.93 (m, 2H), 7.17 (dd, J_1 = 1.6, J_2 = 7.6 Hz, 1H), 7.24 (m, 1H), 9.49 (s, 1H).

^{13}C NMR (125.76 MHz, $CDCl_3$) (ppm): 18.9, 21.8, 48.3, 48.5, 76.4, 128.2, 128.8, 129.2, 135.4, 204.4.

$[\alpha]_D^{25°C}$ = –1.5 (c = 0.4, $CHCl_3$).

HPLC (Chiralcel OD-H, hexane/i-PrOH 80/20, 1 mL/min, λ = 210 nm): t_{minor} = 12.0 min, t_{major} = 17.1 min.

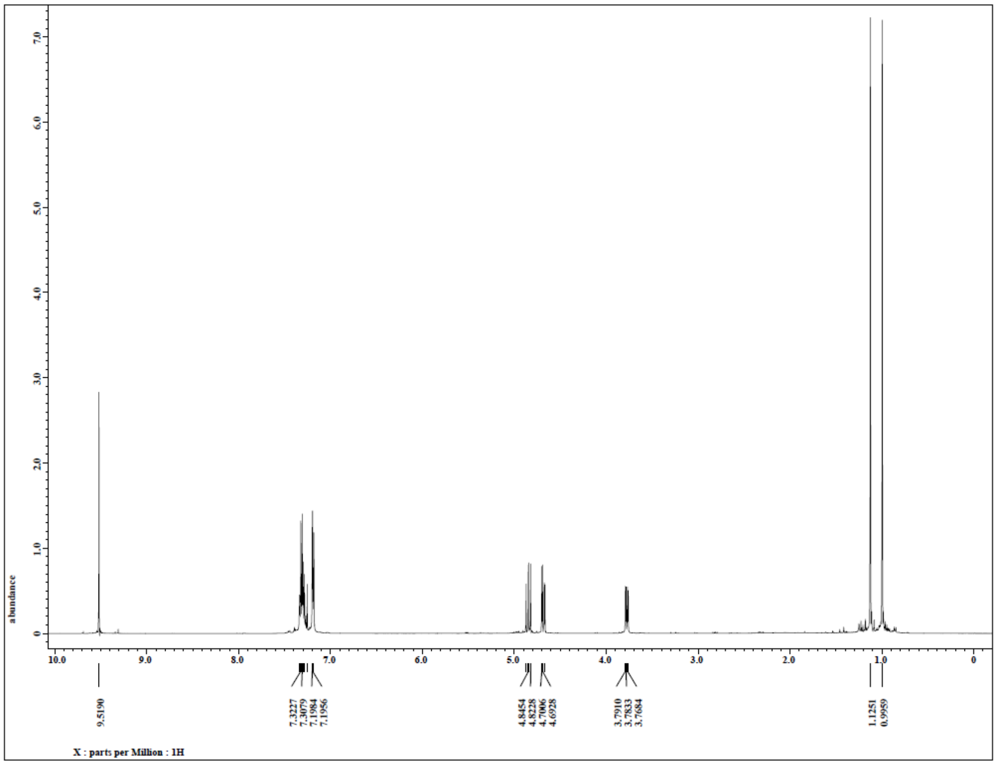

Chromatogram

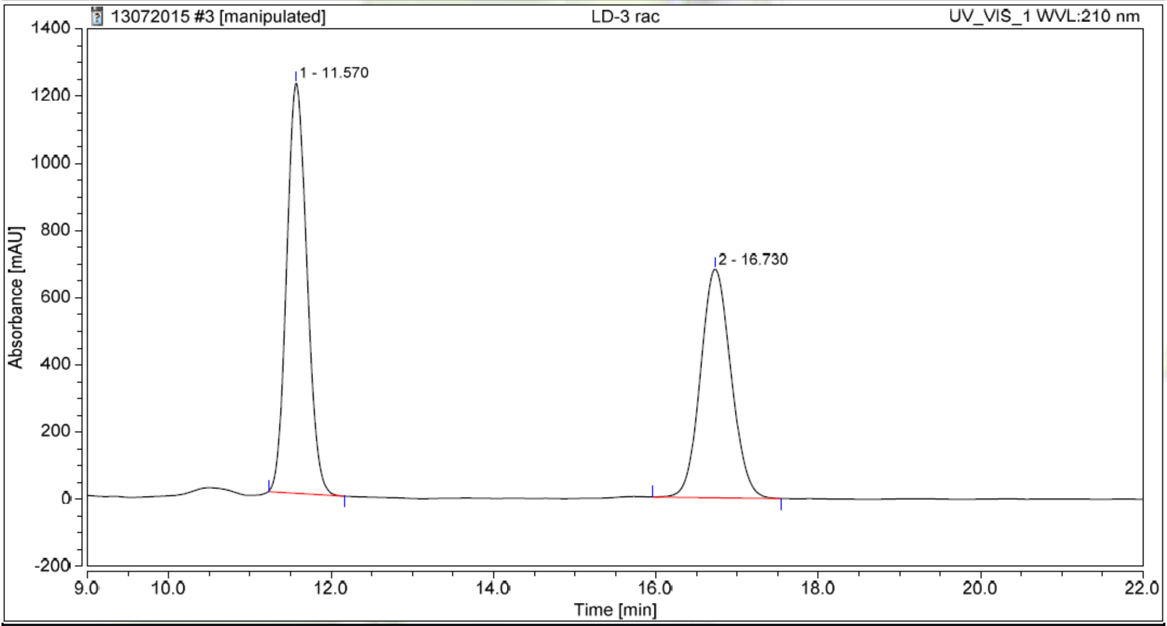

Integration Results

No.	Peak Name	Retention Time<br>min	Area<br>mAU*min	Height<br>mAU	Relative Area<br>%	Relative Height<br>%	Amount<br>n.a.
1		11.570	352.690	1221.508	54.37	64.21	n.a.
2		16.730	295.987	680.785	45.63	35.79	n.a.
**Total:**			**648.677**	**1902.293**	**100.00**	**100.00**	

Chromatogram

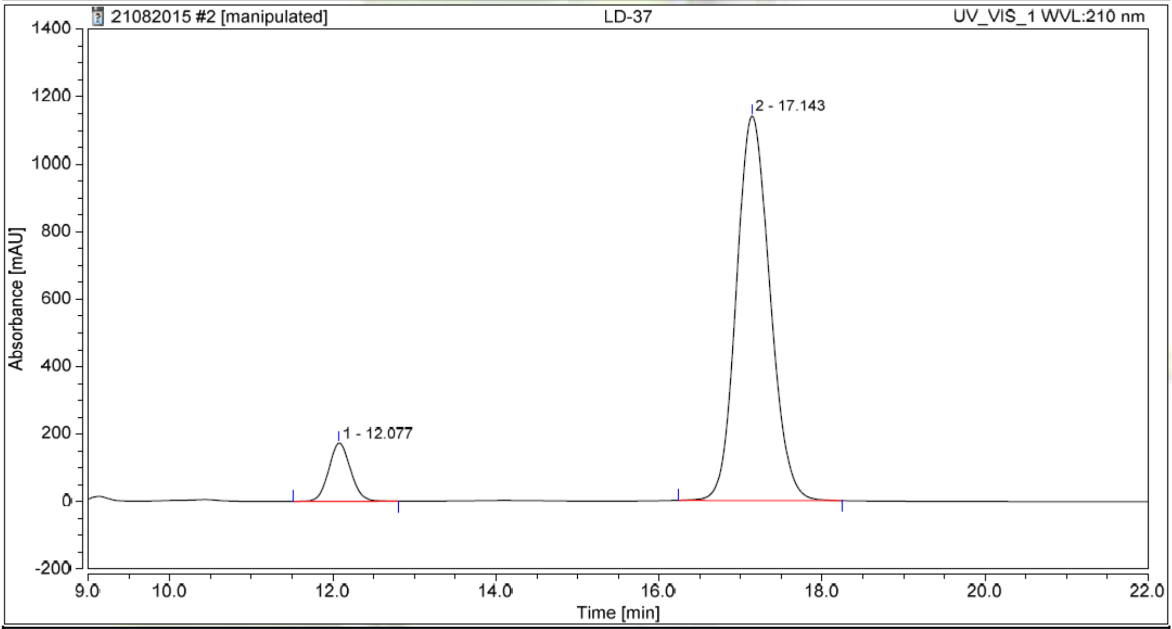

Integration Results

No.	Peak Name	Retention Time min	Area mAU*min	Height mAU	Relative Area %	Relative Height %	Amount n.a.
1		12.077	53.817	173.121	9.01	13.18	n.a.
2		17.143	543.197	1140.264	90.99	86.82	n.a.
**Total:**			**597.014**	**1313.385**	**100.00**	**100.00**	

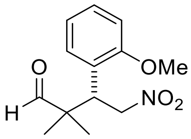

(S)-3-(2-Methoxyphenyl)-2,2-dimethyl-4-nitrobutanal, 15

NMR spectra and HPLC were in agreement with those reported in the literature.[6]

^{1}H NMR (500 MHz, $CDCl_3$) (ppm): 1.03 (s, 3H), 1.08 (s, 3H), 3.80 (s, 3H), 4.23 (a, 1H), 4.71 (dd, J_1 = 4.6, J_2 = 13.1 Hz, 1H), 4.89 (dd, J_1= 10.9, J_2 = 13.1 Hz, 1H), 6.87-7.28 (m, 4H), 9.51 (s, 1H).

^{13}C NMR (125.76 MHz, $CDCl_3$) (ppm): 20.0, 21.1, 48.5, 55.4, 55.4, 75.9, 111.4, 120.8, 124.1, 129.4, 129.8, 157.4, 204.2.

$[\alpha]_D^{25°C}$ = +11.7 (c = 0.47, $CHCl_3$).

HPLC (Chiralcel OD-H, hexane/i-PrOH 80/20, 0.8 mL/min, λ = 210 nm): t_{minor} = 8.9 min., t_{major} = 13.4 min.

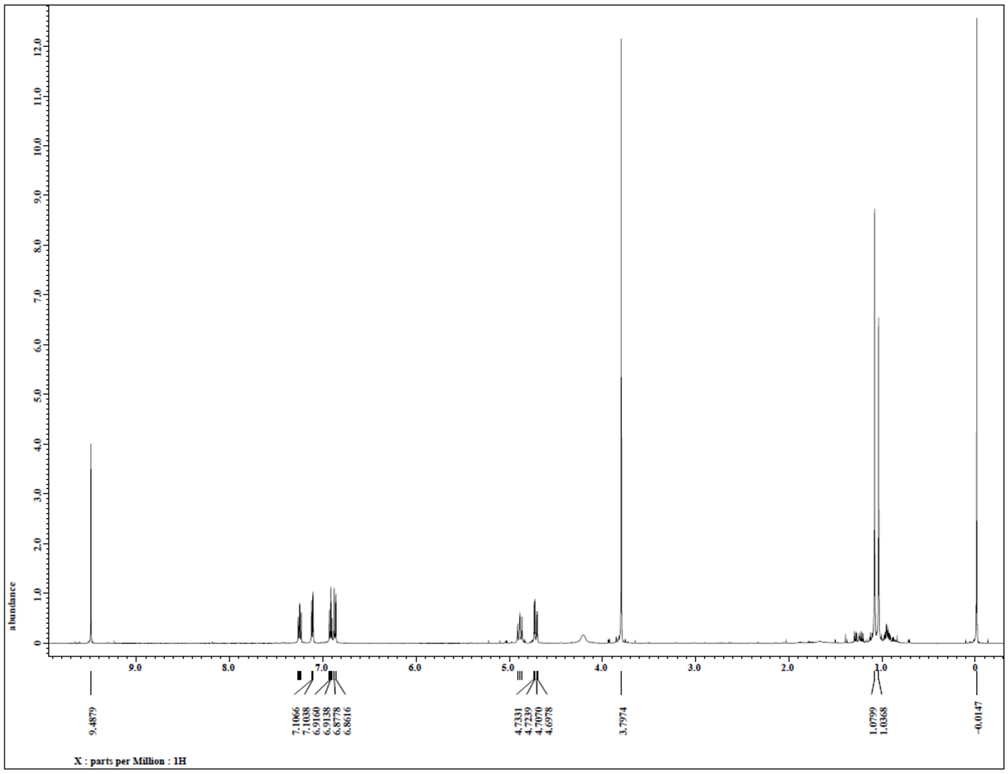

Chromatogram

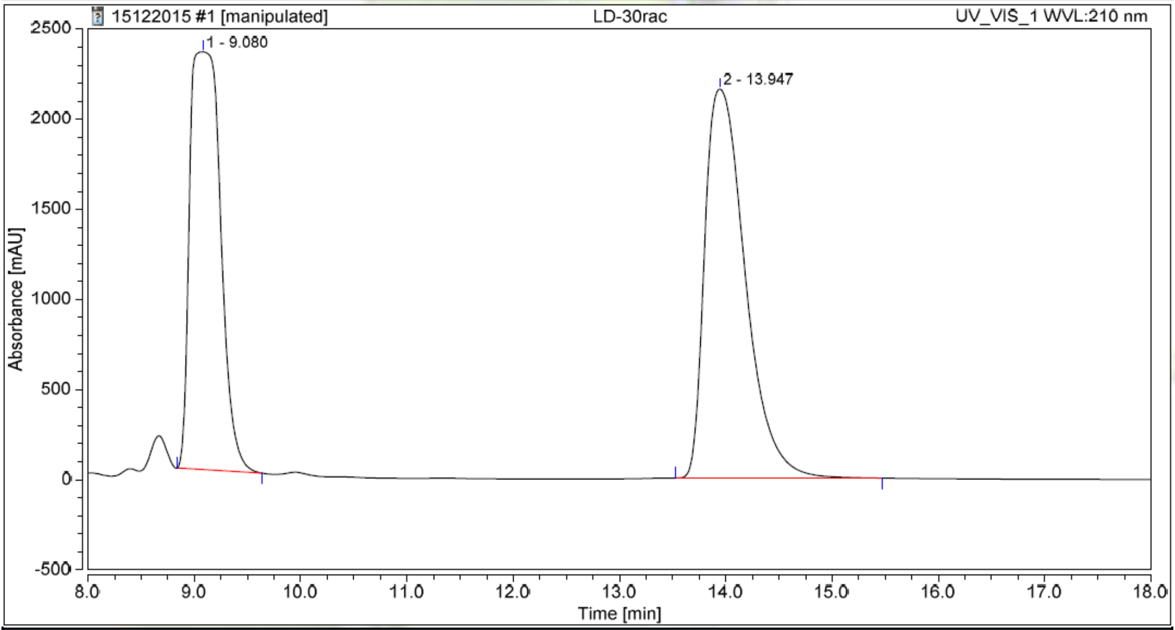

Integration Results

No.	Peak Name	Retention Time min	Area mAU*min	Height mAU	Relative Area %	Relative Height %	Amount n.a.
1		9.080	778.788	2316.848	44.94	51.78	n.a.
2		13.947	954.301	2157.877	55.06	48.22	n.a.
**Total:**			**1733.089**	**4474.726**	**100.00**	**100.00**	

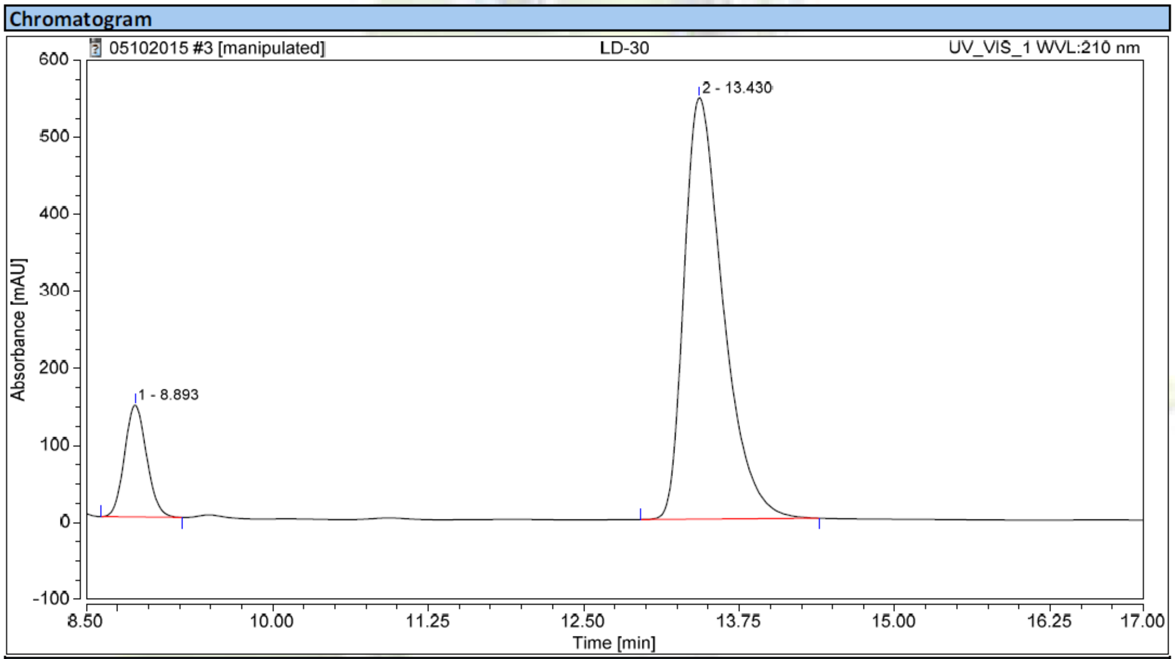

Integration Results

No.	Peak Name	Retention Time min	Area mAU*min	Height mAU	Relative Area %	Relative Height %	Amount n.a.
1		8.893	30.037	145.671	13.13	21.01	n.a.
2		13.430	198.798	547.638	86.87	78.99	n.a.
**Total:**			**228.836**	**693.309**	**100.00**	**100.00**	

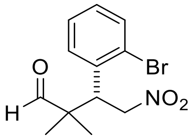

(*R*)-3-(2-Bromophenyl)-2,2-dimethyl-4-nitrobutanal, 16.

Optical rotation was in agreement with that previously reported in the literature.[4] NMR spectra and HPLC were in agreement with those reported in the literature.[6]

^{1}H NMR (500 MHz, $CDCl_3$) (ppm): 1.09 (s, 3H), 1.17 (s, 3H), 4.62 (dd, J_1 = 3.6, J_2 = 11.3 Hz, 1H), 4.72 (dd, J_1 = 4.2, J_2 = 13.6 Hz, 1H), 4.80 (dd, J_1= 11.3, J_2 = 13.1 Hz, 1H), 7.13-7.63 (m, 4H), 9.55 (s, 1H).

^{13}C NMR (125.76 MHz, $CDCl_3$) (ppm): 18.9, 21.1, 45.4, 49.2, 76.5, 127.0, 127.9, 128.4, 129.6, 134.0, 135.6, 203.9.

$[\alpha]_D^{25°C}$ = –0.66 (c = 0.46, $CHCl_3$).

HPLC (Chiralcel OD-H, hexane/i-PrOH 80/20, 1 mL/min, λ = 210 nm): t_{minor} = 10.9 min., t_{major} = 29.0 min.

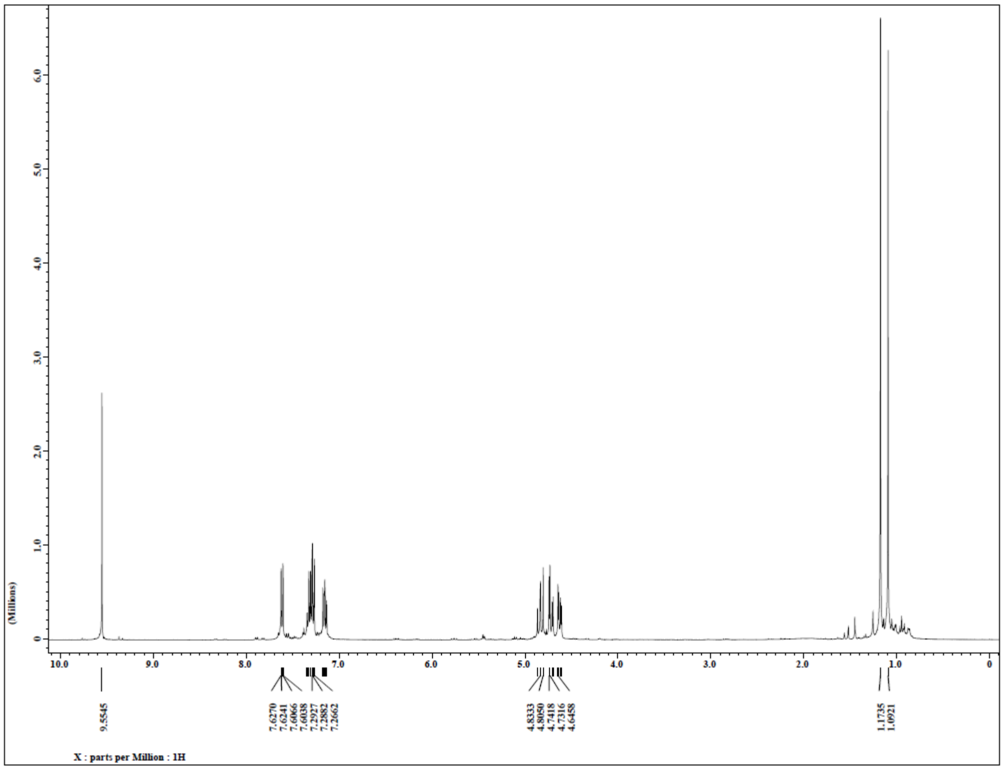

Chromatogram

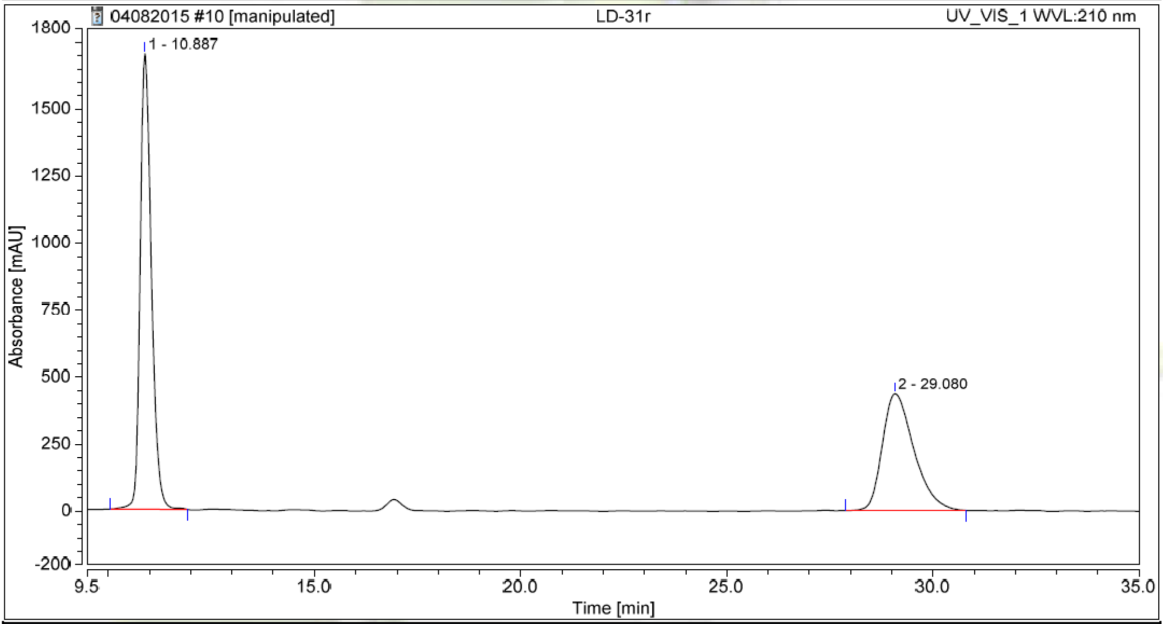

Integration Results

No.	Peak Name	Retention Time min	Area mAU*min	Height mAU	Relative Area %	Relative Height %	Amount n.a.
1		10.887	537.898	1699.530	58.17	79.58	n.a.
2		29.080	386.776	436.025	41.83	20.42	n.a.
**Total:**			**924.674**	**2135.555**	**100.00**	**100.00**	

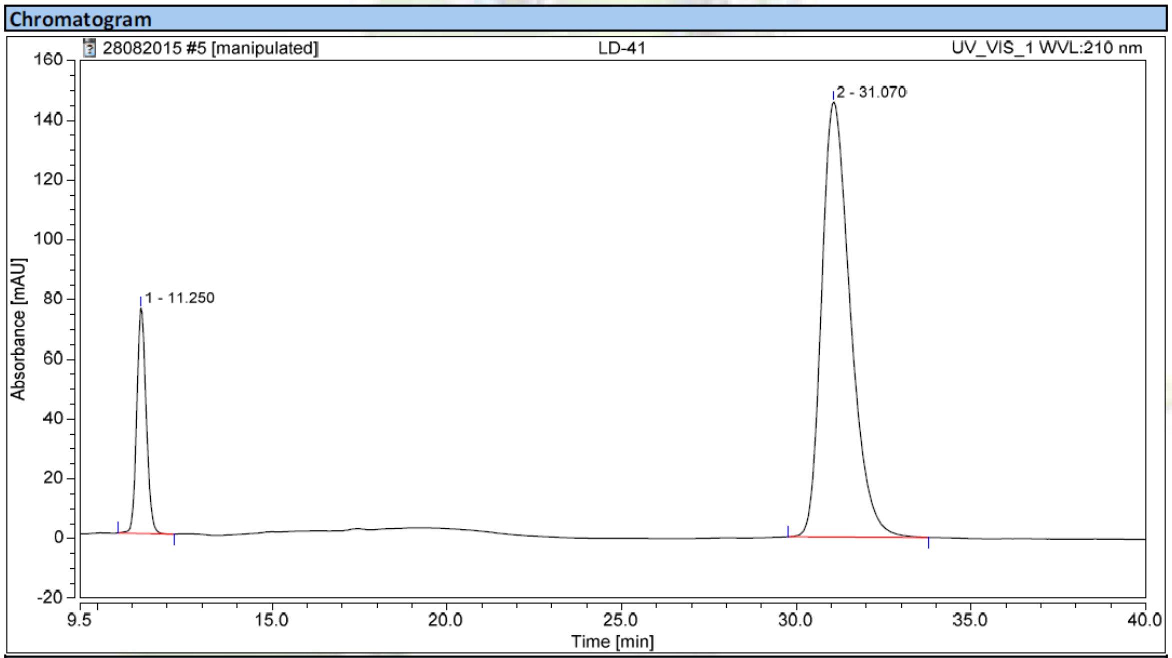

Integration Results

No.	Peak Name	Retention Time min	Area mAU*min	Height mAU	Relative Area %	Relative Height %	Amount n.a.
1		11.250	23.596	75.515	14.54	34.13	n.a.
2		31.070	138.737	145.738	85.46	65.87	n.a.
**Total:**			**162.333**	**221.253**	**100.00**	**100.00**	

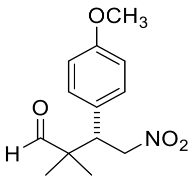

(*S*)-3-(4-Methoxyphenyl)-2,2-dimethyl-4-nitrobutanal, 17.

Optical rotation was in agreement with that previously reported in the literature.[4] NMR spectra and HPLC were in agreement with those reported in the literature.[6]

^{1}H NMR (400 MHz, $CDCl_3$) (ppm): 0.99 (s, 3H), 1.12 (s, 3H), 3.74 (dd, J_1 = 4.2, J_2 = 11.5 Hz, 1H), 3.78 (s, 3H), 4.66 (dd, J_1= 4.3, J_2 = 12.9 Hz, 1H), 4.80 (dd, J_1 = 11.2, J_2 = 12.9 Hz, 1H), 6.85 (d, J_{AB} = 8.6 Hz, 2H), 7.11 (d, J_{AB} = 8.8 Hz, 2H), 9.52 (s, 1H).

^{13}C NMR (100.52 MHz, $CDCl_3$) (ppm): 19.0, 21.7, 47.9, 48.5, 55.3, 76.6, 114.2, 127.2, 130.2, 159.4, 204.6.

$[\alpha]_D^{25°C}$ = +2.7 (c = 0.41, $CHCl_3$).

HPLC (Chiralcel OD-H, hexane/i-PrOH 75/25, 0.8 mL/min, λ = 210 nm): t_{minor} = 13.7 min., t_{major} = 19.9 min.

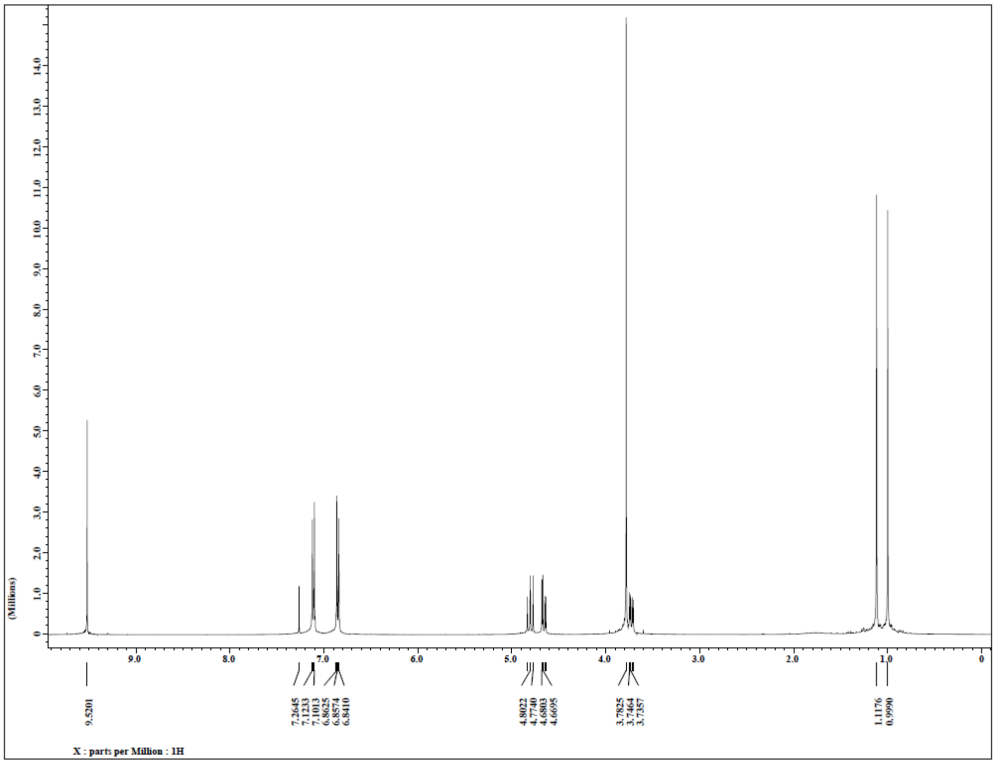

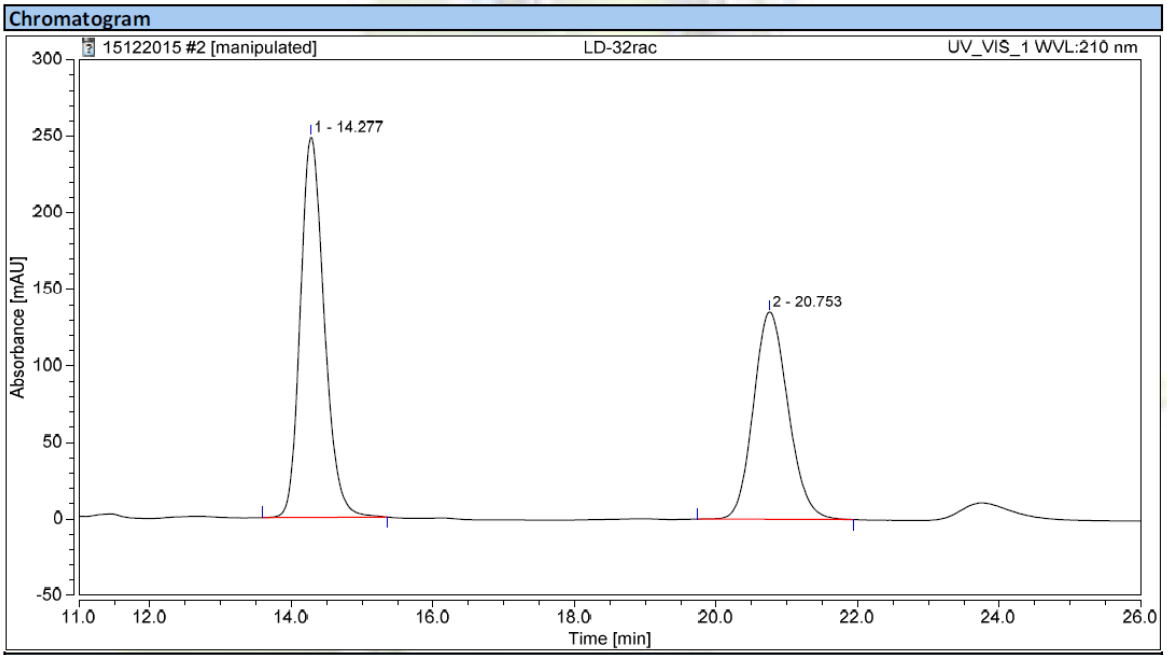

Integration Results

No.	Peak Name	Retention Time min	Area mAU*min	Height mAU	Relative Area %	Relative Height %	Amount n.a.
1		14.277	97.295	248.962	55.84	64.74	n.a.
2		20.753	76.930	135.585	44.16	35.26	n.a.
**Total:**			**174.225**	**384.547**	**100.00**	**100.00**	

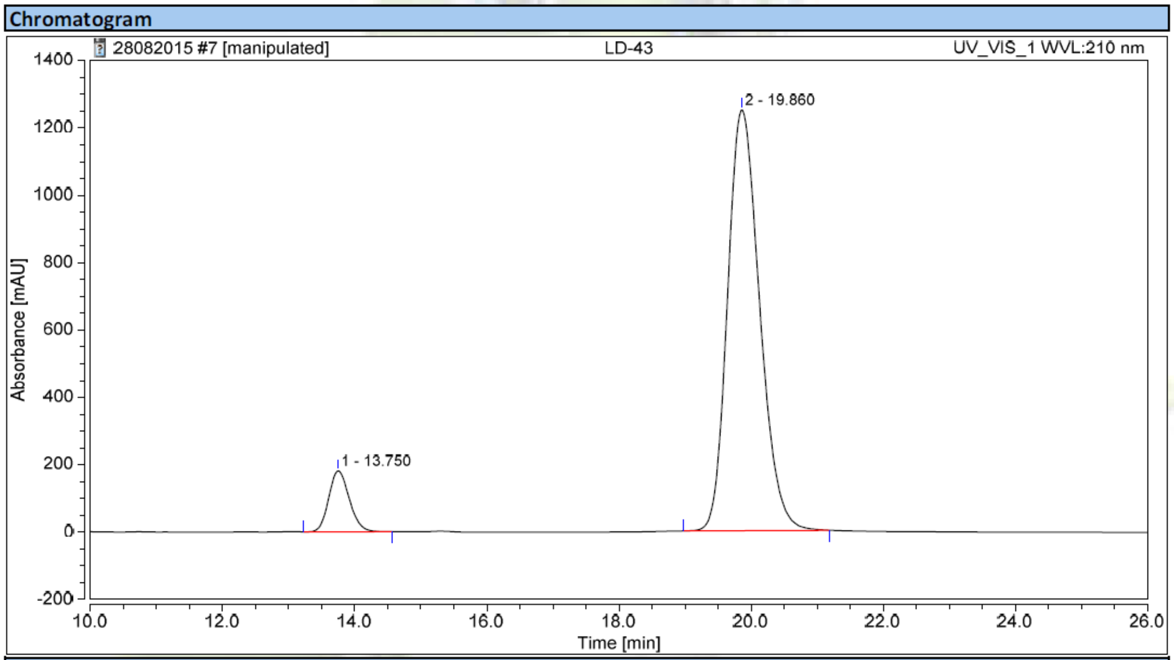

Integration Results

No.	Peak Name	Retention Time min	Area mAU*min	Height mAU	Relative Area %	Relative Height %	Amount n.a.
1		13.750	65.575	181.448	8.54	12.67	n.a.
2		19.860	701.940	1250.514	91.46	87.33	n.a.
**Total:**			**767.515**	**1431.962**	**100.00**	**100.00**	

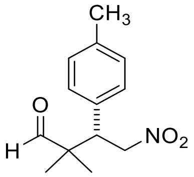

(*S*)-2,2-Dimethyl-4-nitro-3-(*p*-tolyl)butanal, 18.

NMR spectra and HPLC data were in agreement with those reported for the enantiomeric compound in the literature.[6]

^{1}H NMR (500 MHz, $CDCl_3$) (ppm): 0.98 (s, 3H), 1.11 (s, 3H), 2.30 (s, 3H), 3.73 (dd, J_1 = 4.2, J_2 = 11.3 Hz, 1H), 4.65 (dd, J_1= 4.2, J_2 = 12.7 Hz, 1H), 4.81 (dd, J_1 = 11.3, J_2 = 12.7 Hz, 1H), 7.09 (m, 4H), 9.51 (s, 1H).

^{13}C NMR (125.76 MHz, $CDCl_3$) (ppm): 18.9, 21.1, 21.6, 48.2, 48.3, 76.5, 129.0, 129.5, 132.2, 138.0, 204.5.

$[\alpha]_D^{25°C}$ = –1.2 (c = 0.40, $CHCl_3$).

HPLC (Chiralcel OD-H, hexane/i-PrOH 80/20, 1.0 mL/min, λ = 210 nm): t_{minor} = 10.0 min., t_{major} = 14.0 min.

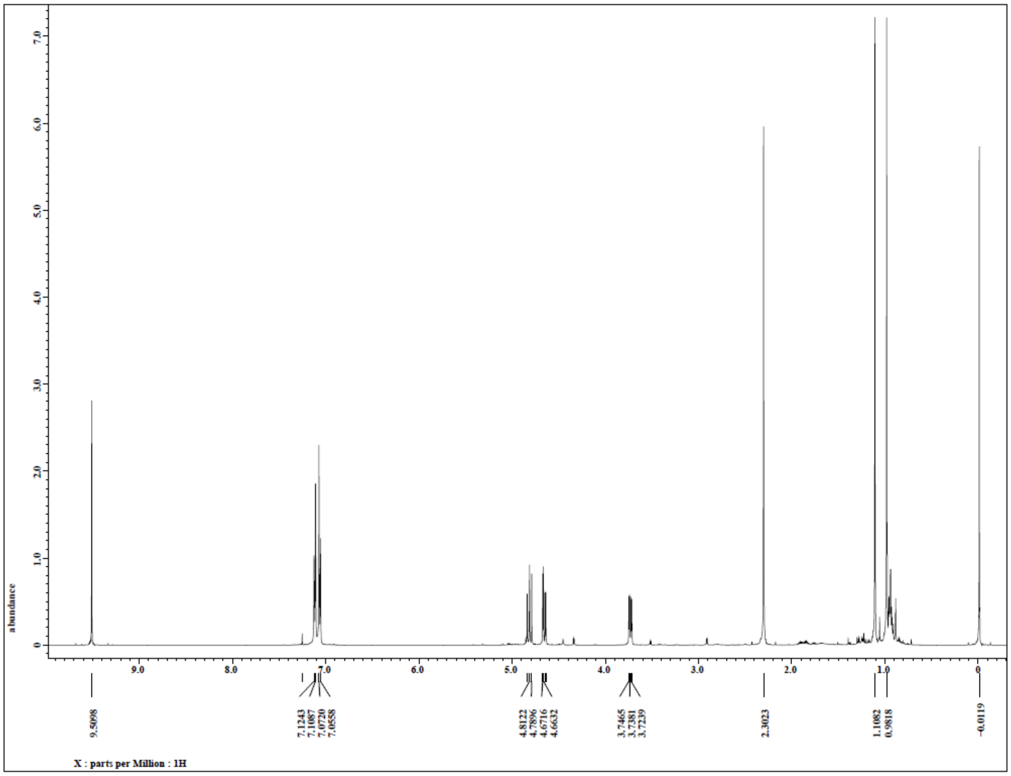

Chromatogram

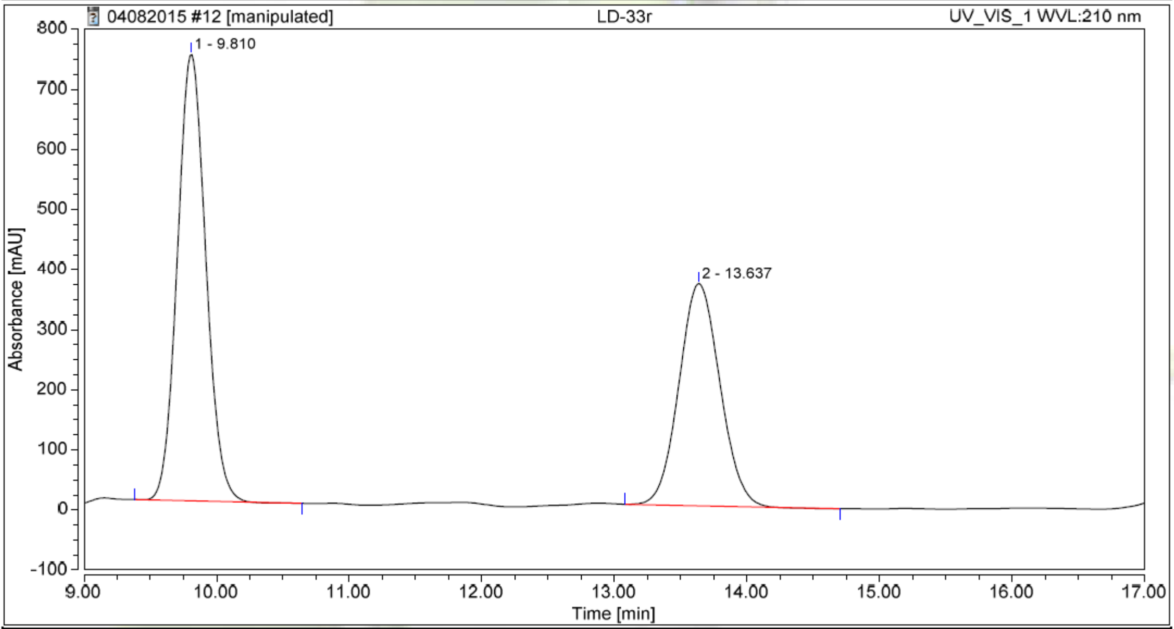

Integration Results

No.	Peak Name	Retention Time<br>min	Area<br>mAU*min	Height<br>mAU	Relative Area<br>%	Relative Height<br>%	Amount<br>n.a.
1		9.810	184.483	743.791	58.26	66.77	n.a.
2		13.637	132.174	370.095	41.74	33.23	n.a.
**Total:**			**316.657**	**1113.886**	**100.00**	**100.00**	

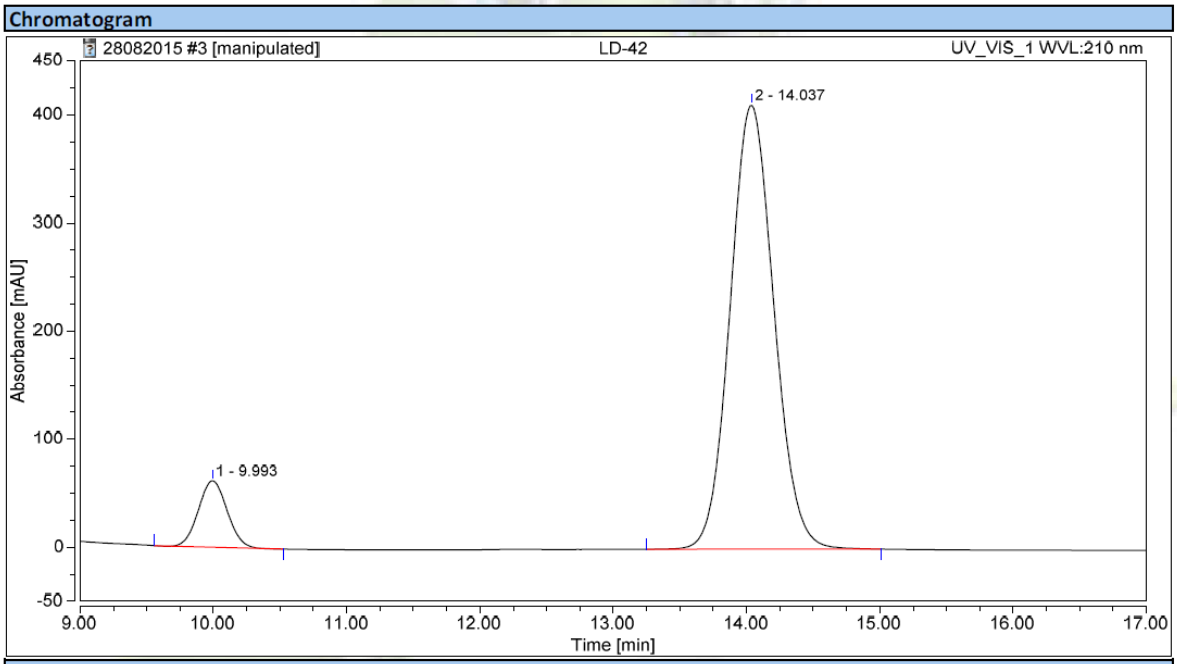

Integration Results

No.	Peak Name	Retention Time min	Area mAU*min	Height mAU	Relative Area %	Relative Height %	Amount n.a.
1		9.993	15.296	61.493	8.92	13.02	n.a.
2		14.037	156.261	410.861	91.08	86.98	n.a.
**Total:**			**171.557**	**472.354**	**100.00**	**100.00**	

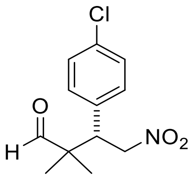

(*S*)-3-(4-Chlorophenyl)-2,2-dimethyl-4-nitrobutanal, 19

NMR spectra and HPLC data in agreement with those reported for the enantiomeric compound in the literature.[6]

^{1}H NMR (500 MHz, $CDCl_3$) (ppm): 1.00 (s, 3H), 1.11 (s, 3H), 3.76 (dd, J_1 = 4.2, J_2 = 11.3 Hz, 1H), 4.68 (dd, J_1= 4.3, J_2 = 13.1 Hz, 1H), 4.81 (dd, J_1 = 11.3, J_2 = 13.1 Hz, 1H), 7.14 (d, J_{AB} = 8.5 Hz, 2H), 7.27 (d, J_{AB} = 8.4 Hz, 4H), 9.49 (s, 1H).

^{13}C NMR (125.76 MHz, $CDCl_3$) (ppm): 19.0, 21.8, 48.0, 48.3, 76.2, 129.1, 130.5, 134.0, 134.3, 203.9.

$[\alpha]_D^{25°C}$ = –3.0 (c = 0.333, $CHCl_3$).

HPLC (Chiralcel OD-H, hexane/i-PrOH 80/20, 0.8 mL/min, λ = 210 nm): t_{minor} = 14.6 min., t_{major} = 23.0 min.

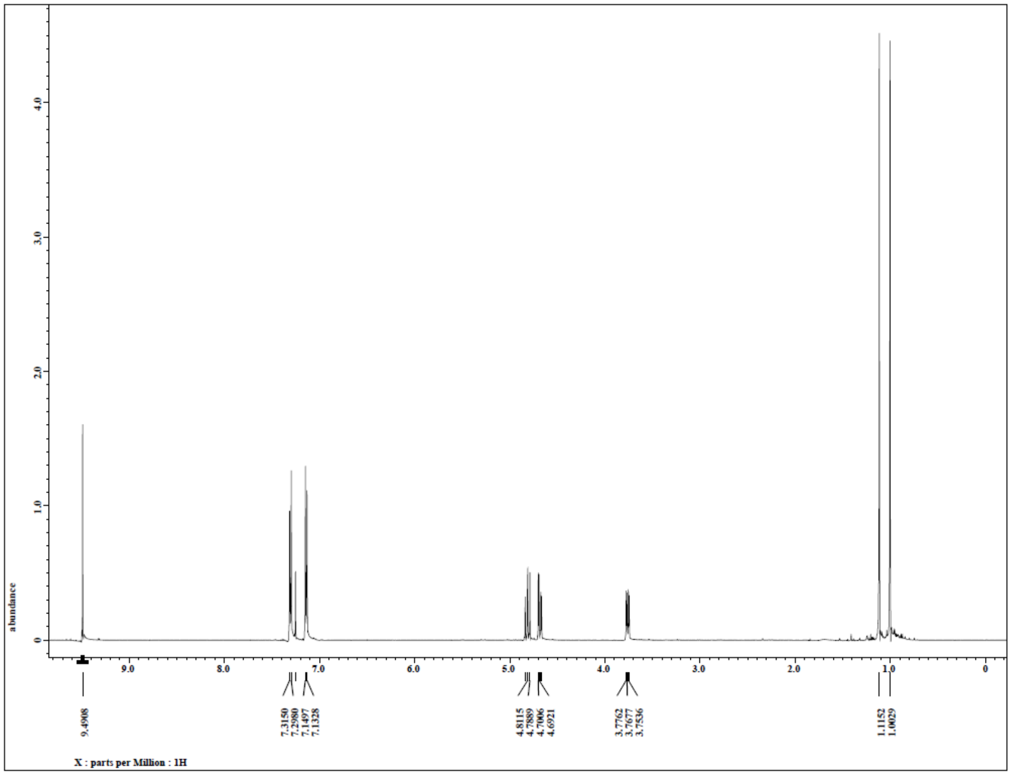

Chromatogram

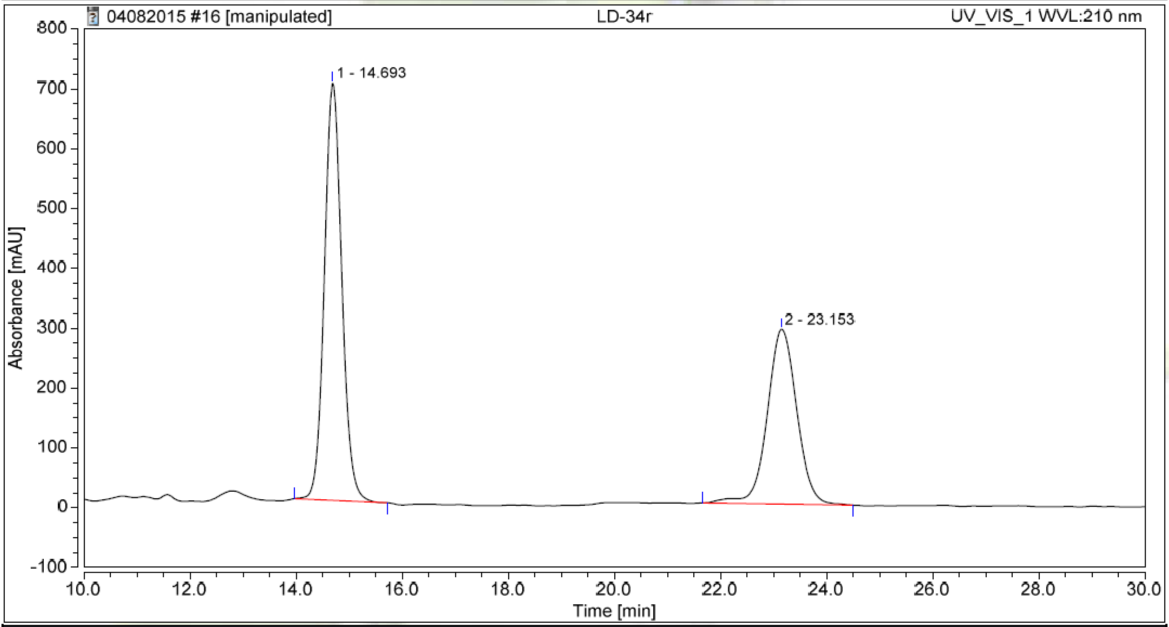

Integration Results

No.	Peak Name	Retention Time min	Area mAU*min	Height mAU	Relative Area %	Relative Height %	Amount n.a.
1		14.693	266.267	697.493	58.49	70.46	n.a.
2		23.153	188.950	292.470	41.51	29.54	n.a.
**Total:**			**455.217**	**989.963**	**100.00**	**100.00**	

Chromatogram

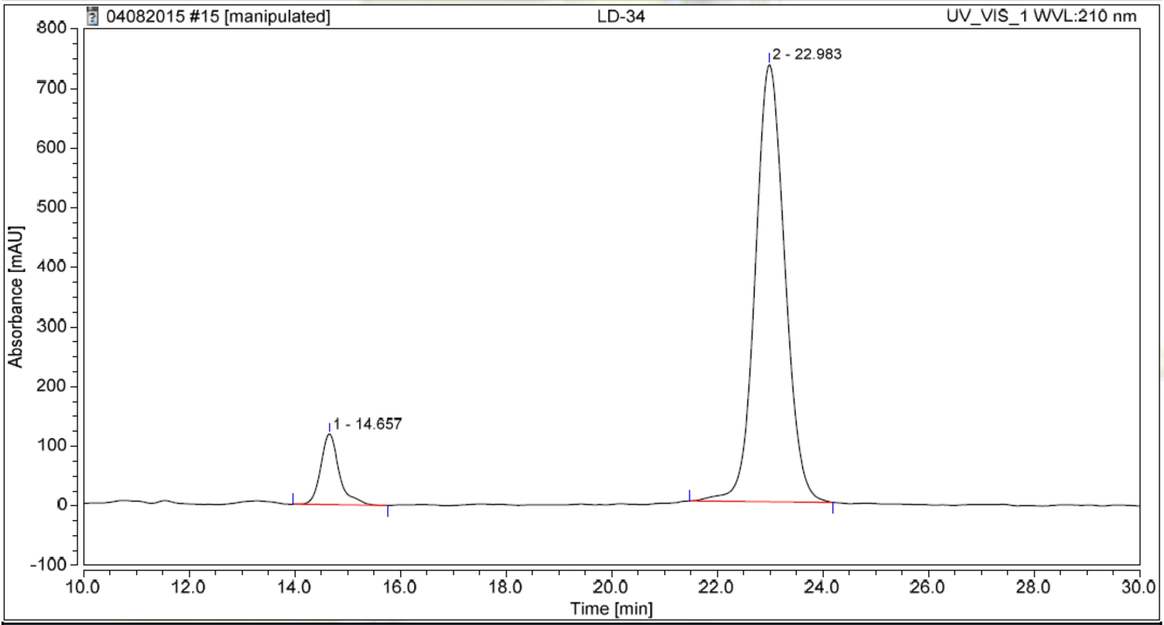

Integration Results

No.	Peak Name	Retention Time min	Area mAU*min	Height mAU	Relative Area %	Relative Height %	Amount n.a.
1		14.657	48.088	119.057	9.19	13.96	n.a.
2		22.983	475.312	733.581	90.81	86.04	n.a.
**Total:**			**523.400**	**852.637**	**100.00**	**100.00**	

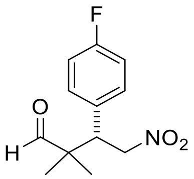

(*S*)-3-(4-Fluorophenyl)-2,2-dimethyl-4-nitrobutanal, 20

NMR spectra and HPLC data were in agreement with those reported in the literature for the enantiomeric compound.[6]

^{1}H NMR (500 MHz, $CDCl_3$) (ppm): 1.00 (s, 3H), 1.16 (s, 3H), 3.77 (dd, J_1 = 4.0, J_2 = 11.6 Hz, 1H), 4.68 (dd, J_1= 3.9, J_2 = 12.9 Hz, 1H), 4.81 (dd, J_1 = 11.6, J_2 = 12.9 Hz, 1H), 7.00-7.19 (m, Hz, 4H), 9.5 (s, 1H).

^{13}C NMR (125.76 MHz, $CDCl_3$) (ppm): 19.0, 21.8, 47.9, 48.3, 76.9, 115.8 (d, $J_{C\text{-}F}$ = 21.1 Hz), 130.8 (d, $J_{C\text{-}F}$ = 8.6 Hz), 131.2, 162.5 (d, $J_{C\text{-}F}$ = 247.6 Hz), 203.9.

$[\alpha]_D^{25°C}$ = +8.0 (c = 0.29, $CHCl_3$).

HPLC (Chiralcel OD-H, hexane/i-PrOH 80/20, 1.0 mL/min, λ = 210 nm): t_{minor} = 10.1 min., t_{major} = 17.2 min.

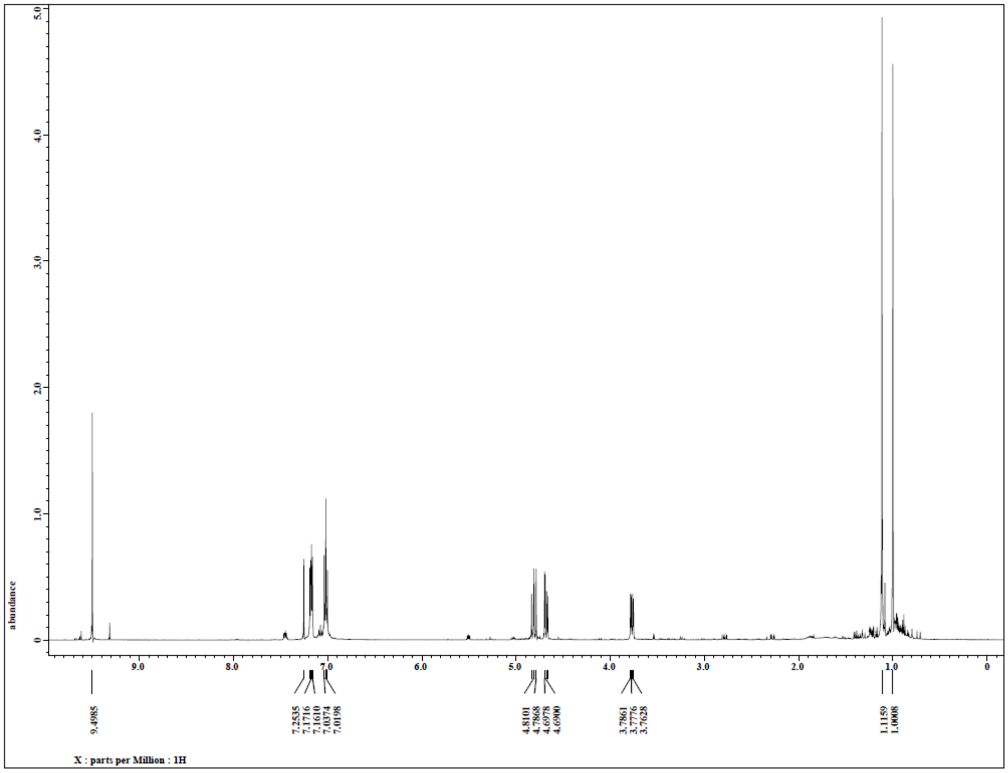

Chromatogram

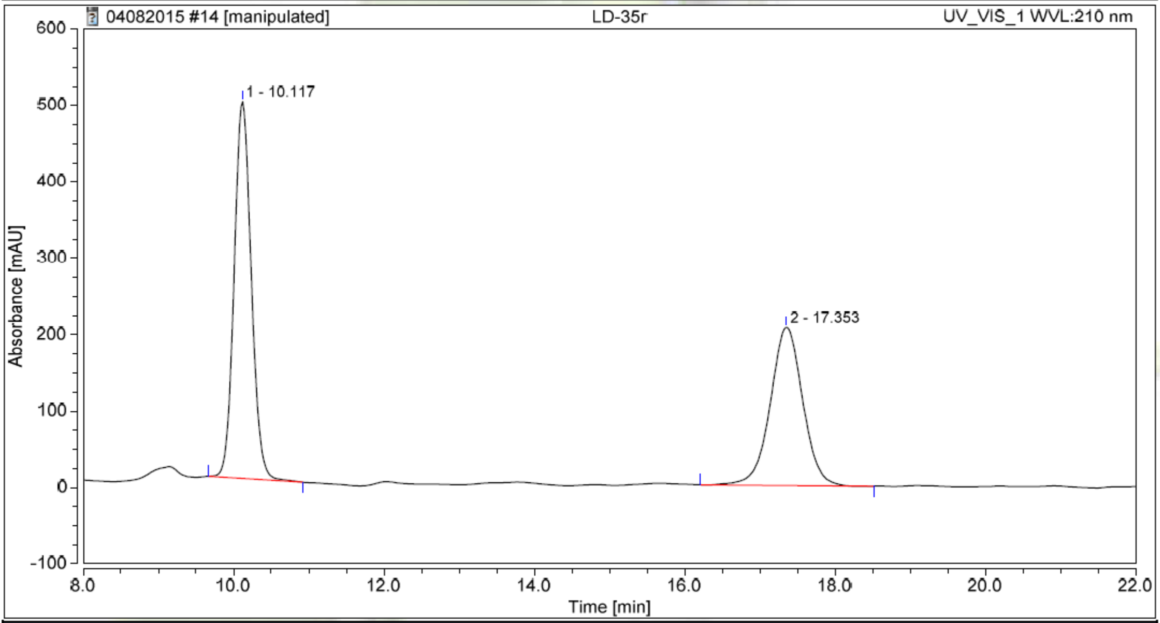

Integration Results

No.	Peak Name	Retention Time min	Area mAU*min	Height mAU	Relative Area %	Relative Height %	Amount n.a.
1		10.117	129.942	493.828	55.76	70.45	n.a.
2		17.353	103.116	207.168	44.24	29.55	n.a.
**Total:**			**233.057**	**700.995**	**100.00**	**100.00**	

Chromatogram

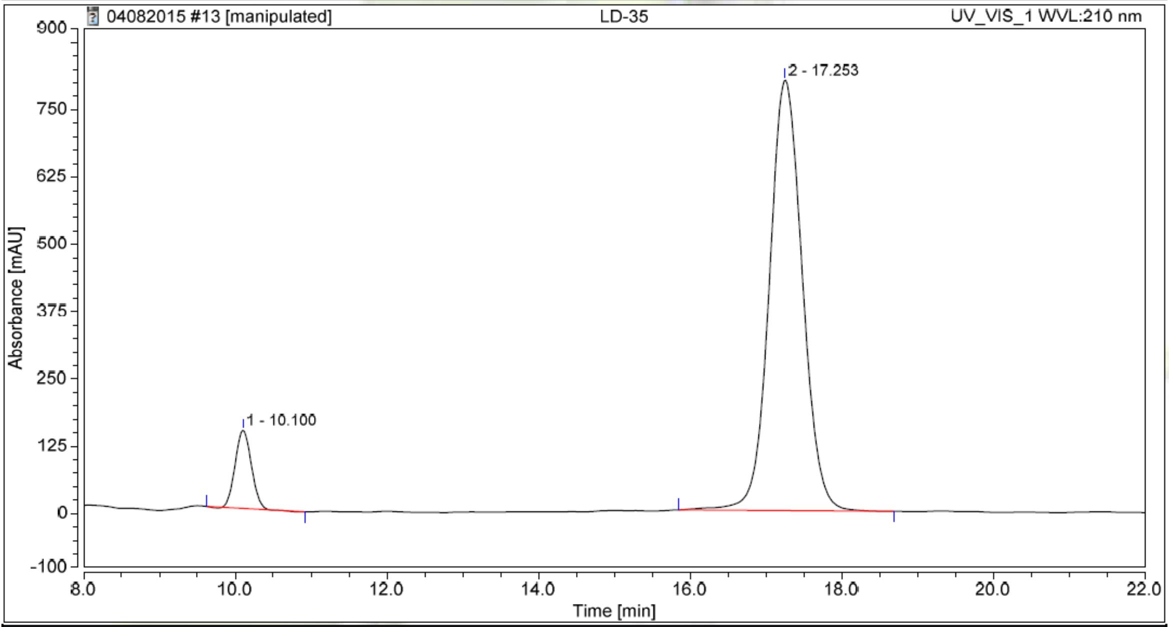

Integration Results

No.	Peak Name	Retention Time min	Area mAU*min	Height mAU	Relative Area %	Relative Height %	Amount n.a.
1		10.100	35.872	145.187	8.16	15.37	n.a.
2		17.253	403.653	799.595	91.84	84.63	n.a.
**Total:**			**439.525**	**944.782**	**100.00**	**100.00**	

4. Bibliography

1. Kokotos, C.G. An asymmetric Michael addition of α,α-disubstituted aldehydes to maleimides leading to a one-pot enantioselective synthesis of lactones catalyzed by amino acids. *Org. Lett.* **2013**, *15*, 2406–2409.
2. Yu, F.; Jin, Z.; Huang, H.; Ye, T.; Liang, X.; Ye, J. A highly efficient asymmetric Michael addition of α,α-disubstituted aldehydes to maleimides catalyzed by primary amine thiourea salt. *Org. Biomol. Chem.* **2010**, *8*, 4767–4774.
3. Avila, A.; Chinchilla, R.; Gómez-Bengoa, E.; Nájera, C. Enantioselective synthesis of succinimides by Michael Addition of aldehydes to maleimides organocatalyzed by chiral primary amine-guanidines. *Eur. J. Org. Chem.* **2013**, *2013*, 5085-5092.
4. Yoshida, M.; Sato, A.; Hara, S. Asymmetric Michael addition of aldehydes to nitroalkenes using a primary amino acid lithium salt. *Org. Biomol. Chem.* **2010**, *8*, 3031-3036.
5. Luo, C.; Du, D.-M. Highly enantioselective Michael addition of ketones and an aldehyde to nitroalkenes catalyzed by a binaphthyl sulfonimide in water. *Synthesis* **2011**, 1968-1973.
6. Nugent, T.C.; Shoaib, M.; Shoaib, A. Practical access to highly enantioenriched quaternary carbon Michael adducts using simple organocatalysts. *Org. Biomol. Chem.* **2011**, *9*, 52–56.
